# Unifying Nonlinear Response and Incoherent Mixing in Action-2D Electronic Spectroscopy

**DOI:** 10.1021/acs.jpclett.3c01670

**Published:** 2023-07-25

**Authors:** Matteo Bruschi, Luca Bolzonello, Federico Gallina, Barbara Fresch

**Affiliations:** † Dipartimento di Scienze Chimiche, Università degli Studi di Padova, via Marzolo 1, Padua 35131, Italy; ‡ ICFO - Institut de Ciencies Fotoniques, The Barcelona Institute of Science and Technology, Castelldefels, Barcelona 08860, Spain; ¶ Padua Quantum Technologies Research Center, Università degli Studi di Padova, Padua 35131, Italy

## Abstract

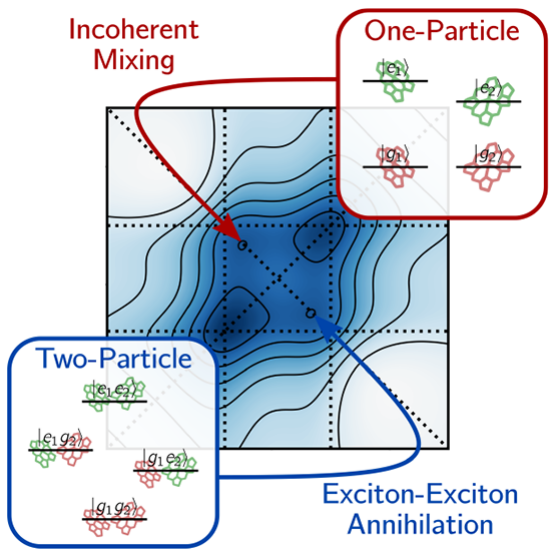

Action-detection has expanded the scope and applicability of 2D electronic spectroscopy, while posing new challenges for the unambiguous interpretation of spectral features. In this context, identifying the origin of cross-peaks at early waiting times is not trivial, and incoherent mixing is often invoked as an unwanted contribution masking the nonlinear signal. In this work, we elaborate on the relation between the nonlinear response and the incoherent mixing contribution by analyzing the action signal in terms of one- and two-particle observables. Considering a weakly interacting molecular dimer, we show how cross-peaks at early waiting times, reflecting exciton–exciton annihilation dynamics, can be equivalently interpreted as arising from incoherent mixing. This equivalence, on the one hand, highlights the information content of spectral features related to incoherent mixing and, on the other hand, provides an efficient numerical scheme to simulate the action response of weakly interacting systems.

The nonlinear optical response triggered by ultrafast laser pulses is the result of a multitude of dynamical processes whose spectral signatures depend specifically on the adopted spectroscopic method. In this context, Two-Dimensional Electronic Spectroscopy (2DES) is the preferred technique to disentangle exciton dynamics of complex systems in both frequency and time domains.^1^ The most prominent version, known as Coherent-2DES (C-2DES), relies on the detection of a coherent signal, emitted along a specific phase-matching direction, upon the interaction with three noncollinear laser pulses.^2,3^

Recently, the technique has been developed to combine the potentialities of 2DES with action-detection schemes.^4,5^ In Action-2DES (A-2DES), the interaction with a train of four collinear laser pulses prepares the system into an excited-state population, which generates an incoherent signal during a long temporal window called the detection time. Due to the collinearity of the setup, the signal contains contributions from various orders in the light–matter interaction. To separate these contributions, the phases of the laser pulses are manipulated through either phase-cycling^6,7^ or phase-modulation^8^ schemes. Depending on the nature of the incoherent signal, different detection schemes have been implemented based on measuring fluorescence,^9−11^ photocurrent,^12−14^ photoions,^15^ and photoelectrons,^16^ paving the way for studying systems in *operando* conditions.^17,18^ Furthermore, the combination of A-2DES with microscopy^19^ and single-molecule^20,21^ techniques allows to go beyond the diffraction limit, thus circumventing the effects of inhomogeneous disorder.

Although probing the same ultrafast dynamics, action-detected spectra exhibit significant differences compared to those obtained through coherent detection. In fact, it was early recognized that spectral features in A-2DES are determined not only by the coherent dynamics induced by the light–matter interaction but also by the dynamical processes taking place during the detection time.^22^ Contrary to C-2DES,^23,24^ the presence of cross-peaks at early waiting times does not represent a univocal signature of excitonic delocalization in A-2DES,^10,25^ having been reported even in the case of weakly interacting systems.^11,19^ By analyzing the different contributions to the response function for a molecular dimer, Malý and Mančal demonstrated that cross-peaks can emerge from the incomplete cancellation of different pathways as a consequence of exciton–exciton annihilation during the detection time.^26^ Following the same line, several other contributions highlighted the importance of two-exciton manifold dynamics in determining spectral features.^27−30^

On the other hand, Grégoire et al. brought to the attention the phenomenon of incoherent mixing as an unwanted contribution in A-2DES spectra.^31^ Incoherent mixing occurs from the combination of linear signals due to nonlinear population dynamics,^31^ e.g., exciton–exciton annihilation, bimolecular recombination, and Auger recombination, or due to nonlinearities in the detection process.^32^ Since incoherent mixing can hide spectral features of the coherent nonlinear response, efforts have been devoted to distinguishing these two contributions. In a theoretical analysis of the action signal, Kalaee et al. proposed the existence of a precise phase relationship between the “true” nonlinear response and incoherent mixing signals, which can be used to differentiate them.^33^

The appearance of cross-peaks at early waiting times and the phenomenon of incoherent mixing have always been considered independently in the literature about A-2DES. The aim of this work is to propose a unifying picture of these two aspects by demonstrating that, beyond sharing a common origin, they actually represent two different views of the same dynamical process when considering a system composed of weakly interacting units.

After a brief presentation of the A-2DES technique, we discuss in detail the case of a pair of chromophores, although the analysis can be generalized to more complex interaction networks. To this end, we use one- and two-particle representations^29,34^ as interpretative tools, where the term “particle” refers to a chromophore in our model. By employing Feynman Diagrams (FDs), the optical response of the chromophoric pair is rationalized using both representations, thereby elucidating the pathways followed by the system during coherent excitation. Since the contribution of the pathways to the total spectrum also depends on the processes occurring during the detection time, we formulate the dynamics in terms of kinetic schemes for one- and two-particle populations. Interestingly, the resulting signal from one-particle populations evidences the net contribution of incoherent mixing stemming from a set of Feynman diagrams where the two pairs of pulses interact with different chromophores. Therefore, we demonstrate how the presence of cross-peaks at early waiting times in A-2DES of weakly interacting systems can be interpreted as arising either from the imperfect cancellation of Feynman pathways (two-particle perspective) or from incoherent mixing (one-particle perspective) as a result of the detection time dynamics. The contribution of the dynamics-induced nonlinearities to the spectrum depends on the specific kinetics of the energy redistribution during the detection time. This implies, on the one hand, that the phase of the incoherent mixing signal is not *a priori* different from that of the nonlinear response and, on the other hand, that the study of incoherent mixing spectral features is informative of dynamical processes in weakly interacting systems. Furthermore, the analysis of the action signal in terms of one-particle observables provides an advantageous computational scheme to simulate the effects of the detection time dynamics in the A-2DES spectra of weakly interacting systems by solving a set of dynamical equations scaling linearly with the number of chromophores.

In A-2DES, the system interacts with a train of four collinear laser pulses, separated by delay times *T*_1_, *T*_2_, and *T*_3_, resulting in the emission of an incoherent signal  during the detection time *T*_*d*_ (Figure 1a). Typically, signal emission in A-2DES is not time-resolved, and the experimentally accessible observable is represented by the time-integrated signal along the detection time *T*_*d*_:
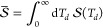
1By adopting a phase-modulation scheme,^8^ the phase of the *i*-th pulse is linearly modulated, from one train to the following, as Φ(Ω_*i*_) = 2*πΩ*_*i*_*mT*, where Ω_*i*_ is the modulation frequency, *m* is the repetition index of the train, and *T* is the intertrain delay time. As a consequence, the incoherent signal itself is modulated and it can be decomposed as:
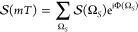
2where  is the component of the signal modulated at the linear combination of frequencies Ω_*S*_ = , where  = 0, ±1, ±2, etc. By taking the Fourier transform along *mT*, the different components of the optical response can be extracted, i.e., rephasing (Ω_*R*_ = −Ω_1_ + Ω_2_ + Ω_3_ – Ω_4_), non-rephasing (Ω_*NR*_ = +Ω_1_ – Ω_2_ + Ω_3_ – Ω_4_) and double-quantum coherence (Ω_*DQC*_ = +Ω_1_ + Ω_2_ – Ω_3_ – Ω_4_) signals. By scanning the different delay times *T*_1_, *T*_2_, and *T*_3_ and taking the Fourier transform along *T*_1_ and *T*_3_, a 2D spectrum is obtained as a function of ℏω_1_ and ℏω_3_ for each value of waiting time *T*_2_.

**Figure 1:**
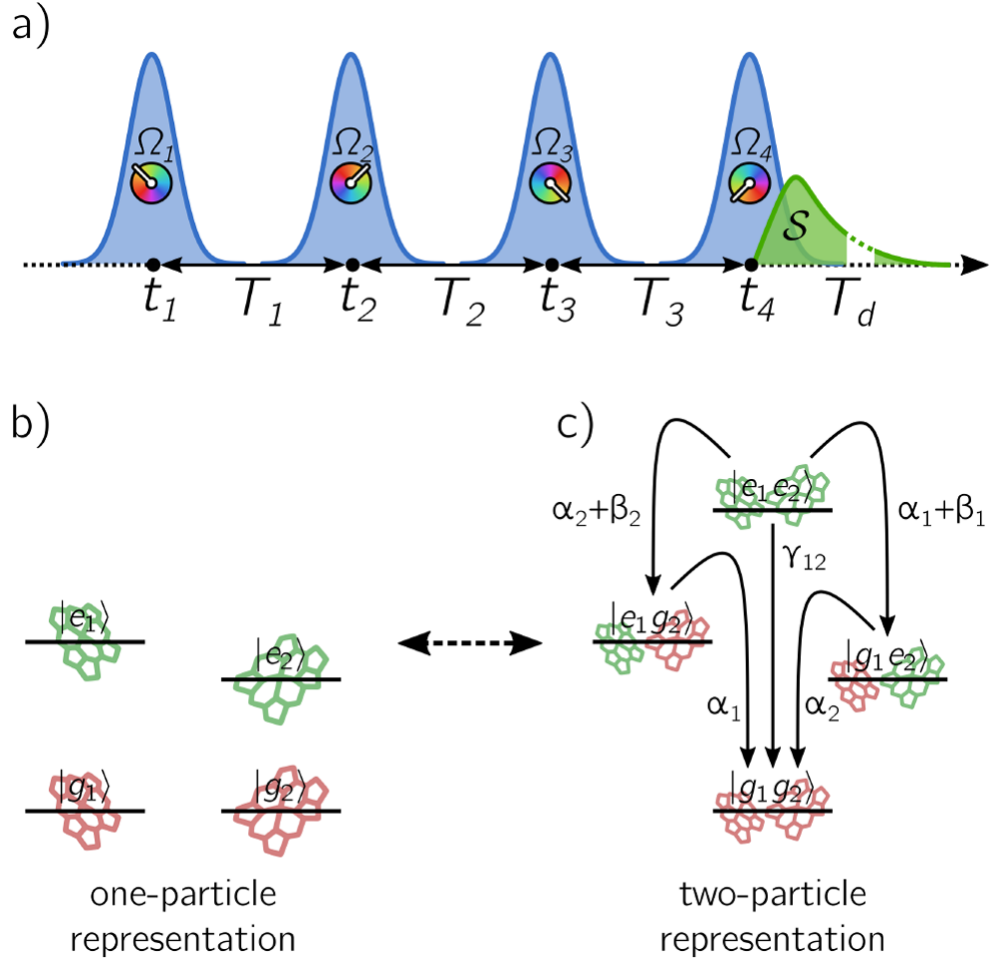
(a) Train of four collinear laser pulses, separated by delay times *T*_1_, *T*_2_, and *T*_3_, whose phases are modulated at frequencies Ω_*i*_, for *i* = 1, ..., 4. As a result of the light–matter interaction, the system emits an incoherent signal  during the detection time *T*_*d*_. States of a weakly interacting pair of chromophores in the (b) one- and (c) two-particle representations, along with the kinetic scheme for populations: α_*n*_ is the rate of exciton recombination, while β_*n*_ and γ_12_ are the rates of exciton–exciton annihilation of one and two excitons, respectively.

This procedure can be numerically implemented in close analogy with the experiment. By employing a non-perturbative treatment of the light–matter interaction, the dynamics of the system is modeled using the Lindblad quantum master equation.^30,35,36^ Details of the computational procedure and the parameters used for the simulations are reported in the Supporting Information.

Let us consider a pair of chromophores, each treated as a two-level electronic system with a ground- |*g*_*n*_⟩ and an excited-state |*e*_*n*_⟩, where the index *n* = 1, 2 denotes the *n*-th molecule (Figure 1b,c). The chromophoric pair is described by the Hamiltonian:
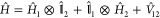
3where *Ĥ*_*n*_ = ϵ_*n*_ |*e*_*n*_⟩⟨*e*_*n*_| and  are the Hamiltonian and the identity operator of the *n*-th chromophore, respectively, while *V̂*_12_ is the excitonic coupling between them. The excitation energies ϵ_*n*_ of the two chromophores are chosen to match those of the B800 and B850 rings of the LH2 complex, namely, ϵ_1_ = 1.55 eV and ϵ_2_ = 1.46 eV.^35^ In the following, the excitonic coupling *V̂*_12_ is assumed to be small such that the two chromophores are weakly interacting. The assumption of weak interaction implies that the eigenstates of the chromophoric pair are well approximated by the product of single chromophore states, whereas the dynamical effects of the interaction are captured at the level of perturbation theory in the form of incoherent transfer rates related to Exciton Energy Transfer (EET) and Exciton–Exciton Annihilation (EEA) processes.^37−39^

Let us now introduce one- and two-particle representations of the system, as proposed by Mukamel^34^ and Kühn et al.^29^ In the one-particle representation (Figure 1b), the state of one chromophore is addressed independently of the other, as described by one-particle observables, e.g., one-particle populations *P*_*e*_1__(*t*) and *P*_*e*_2__(*t*) representing the probability that one chromophore is excited. Although the one-particle observables are well-defined at every time, the presence of interactions between the two chromophores requires a two-particle representation (Figure 1c) of the system, in which the state of both chromophores is simultaneously considered in terms of two-particle observables, e.g., two-particle populations *P*_*e*_1_*g*_2__(*t*) and *P*_*g*_1_*e*_2__(*t*), representing the joint probabilities of one chromophore being excited while the other is in the ground state, and *P*_*e*_1_*e*_2__(*t*), representing the probability that both chromophores are excited. Indeed, in EET the excitation is transferred from one chromophore in the first excited-state to the other in the ground-state. Instead, EEA is a two-step process that is possible only when both chromophores are simultaneously excited: first, a higher excited-state is generated on one molecule leaving the other in the ground-state, and then, rapid internal conversion to the first excited-state takes place, resulting in the net loss of one exciton. Alternatively, the EEA process may result in the annihilation of both excitons. By definition, the two representations are related by *P*_*e*_1__(*t*) = *P*_*e*_1_*g*_2__(*t*) + *P*_*e*_1_*e*_2__(*t*) and *P*_*e*_2__(*t*) = *P*_*g*_1_*e*_2__(*t*) + *P*_*e*_1_*e*_2__(*t*).

The time-resolved incoherent signal is proportional to the two-particle populations weighted by the emission rate of the states:
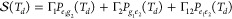
4In general, the different nature of the multiexciton state can be captured by assuming Γ_12_ ≠ Γ_1_ + Γ_2_.^13^ However, when the constraint Γ_12_ = Γ_1_ + Γ_2_ applies, the signal can be expressed equivalently in terms of one-particle populations:
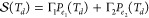
5Due to phase modulation, excited-state populations are modulated, leading to the decomposition of the incoherent signal in eq 2.

Because of the weak interaction between the chromophores, we now assume a net separation in the time scales of the system dynamics. The first time scale is ruled by the interaction with the laser pulses, which probes the coherent dynamics of the system in the range of hundreds of femtoseconds, for short waiting times *T*_2_. On such a time scale, the occurrence of EET and EEA can be neglected. In contrast, the detection time *T*_*d*_ defines a slower time scale, in the nanosecond regime, dictated by the relaxation dynamics at the origin of the incoherent signal. In this case, both EEA and EET processes must be considered.

Although several components of the optical response are readily available from the nonperturbative simulation, in the following, we specifically focus on the rephasing signal , at waiting time *T*_2_ = 0 fs. Considerations for the non-rephasing and double-quantum coherence signals are drawn in the Supporting Information.

Before discussing the role of the dynamical evolution during the detection time, we first consider the contributions to the spectrum resulting from populations at *T*_*d*_ = 0 fs, immediately after the end of the fourth pulse. This is equivalent to assuming signal emission as the only relaxation pathway active during the detection time. In Figure 2, we report the contributions to the signals in eqs 4 and 5 from two- (Figure 2a–c) and one-particle populations (Figure 2d,e), respectively. As exemplified in Figure 2f, spectral features for the considered system may appear either as diagonal peaks (D1 and D2) or as cross-peaks (C1 and C2).

**Figure 2:**
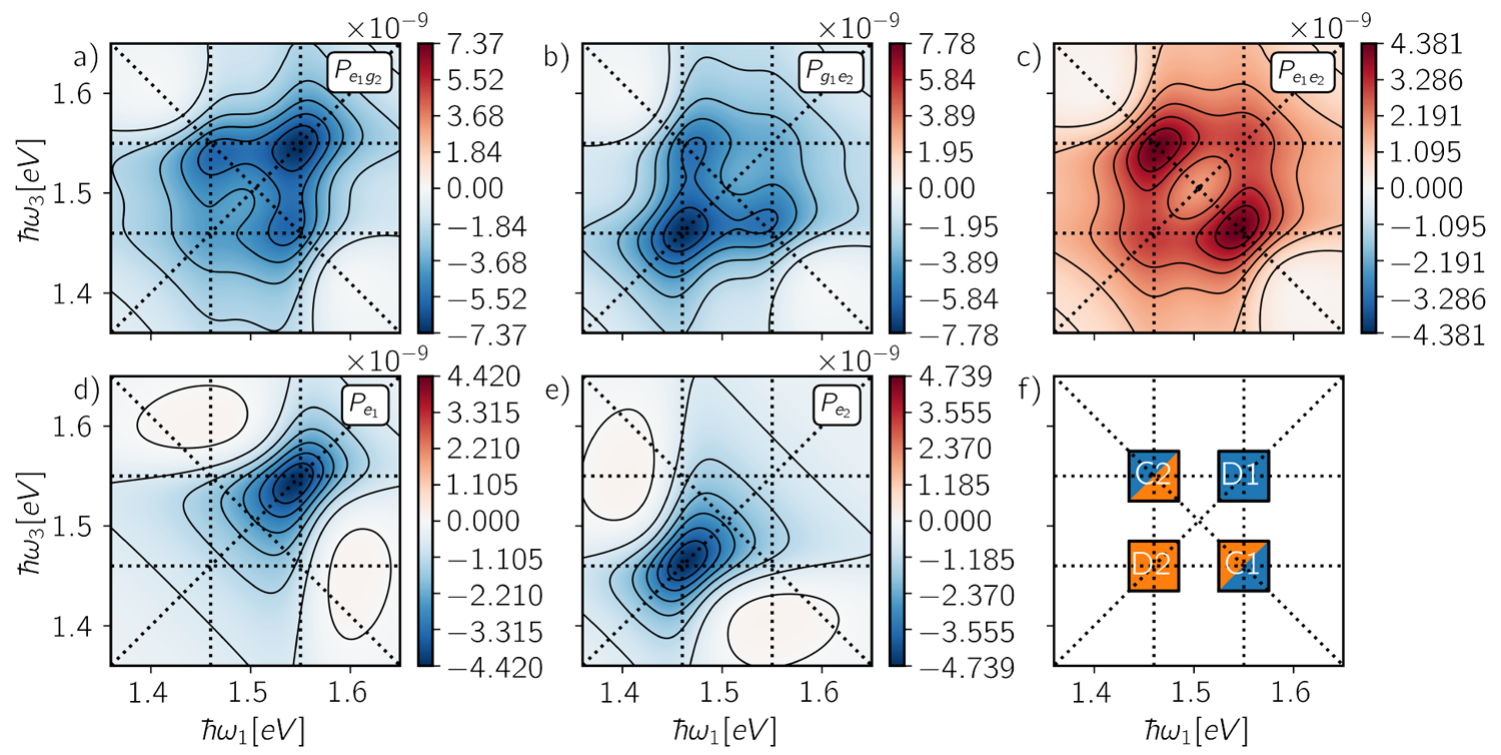
Rephasing spectra from two-particle populations (a) *P*_*e*_1_*g*_2__, (b) *P*_*g*_1_*e*_2__, and (c) *P*_*e*_1_*e*_2__ and one-particle populations (d) *P*_*e*_1__ and (e) *P*_*e*_2__ at detection time *T*_*d*_ = 0 fs. In (f) are reported the spectral positions of diagonal peaks (D1 and D2) and cross-peaks (C1 and C2).

The spectra from two-particle populations *P*_*e*_1_*g*_2__ and *P*_*g*_1_*e*_2__ exhibit a diagonal peak and two cross-peaks of negative sign, whereas *P*_*e*_1_*e*_2__ displays two positive cross-peaks. In this context, the cross-peaks from two-particle populations represent statistical correlations between the chromophores induced by the light–matter interaction, rather than an actual coupling between them.^29,34^ Indeed, when partitioning the signal in terms of one-particle populations *P*_*e*_1__ and *P*_*e*_2__, the cross-peaks from the two-particle populations cancel out completely, and each spectrum exhibits a negative diagonal peak corresponding to the response of an independent molecule, as expected for a pair of weakly interacting chromophores at short waiting times *T*_2_.

The cancellation of cross-peaks that is observed when switching from the two- to one-particle representation relies on the specific phase relation between different excitation pathways. These pathways can be visualized in terms of Feynman diagrams corresponding to Ground-State Bleaching (GSB), Stimulated Emission (SE), and Excited-State Absorption (ESA) contributions.^40^ Due to the presence of a fourth pulse, two kinds of ESA pathways are possible in A-2DES: generating either a one-exciton population (ESAI) or a two-exciton population (ESAII).^22^ Each FD contributes to the signal with the sign (−1)^*n*_B_^, where *n*_B_ is the number of interactions on the *bra* side. A selection of FDs contributing to the optical response of the system is shown in Figure 3, while the complete set is given in the Supporting Information along with the corresponding response functions. Notice that also FDs can be represented in terms of one- (1P-FD) and two-particle (2P-FD) observables,^29,41^ as reported respectively on the right and left of each panel in Figure 3.

**Figure 3:**
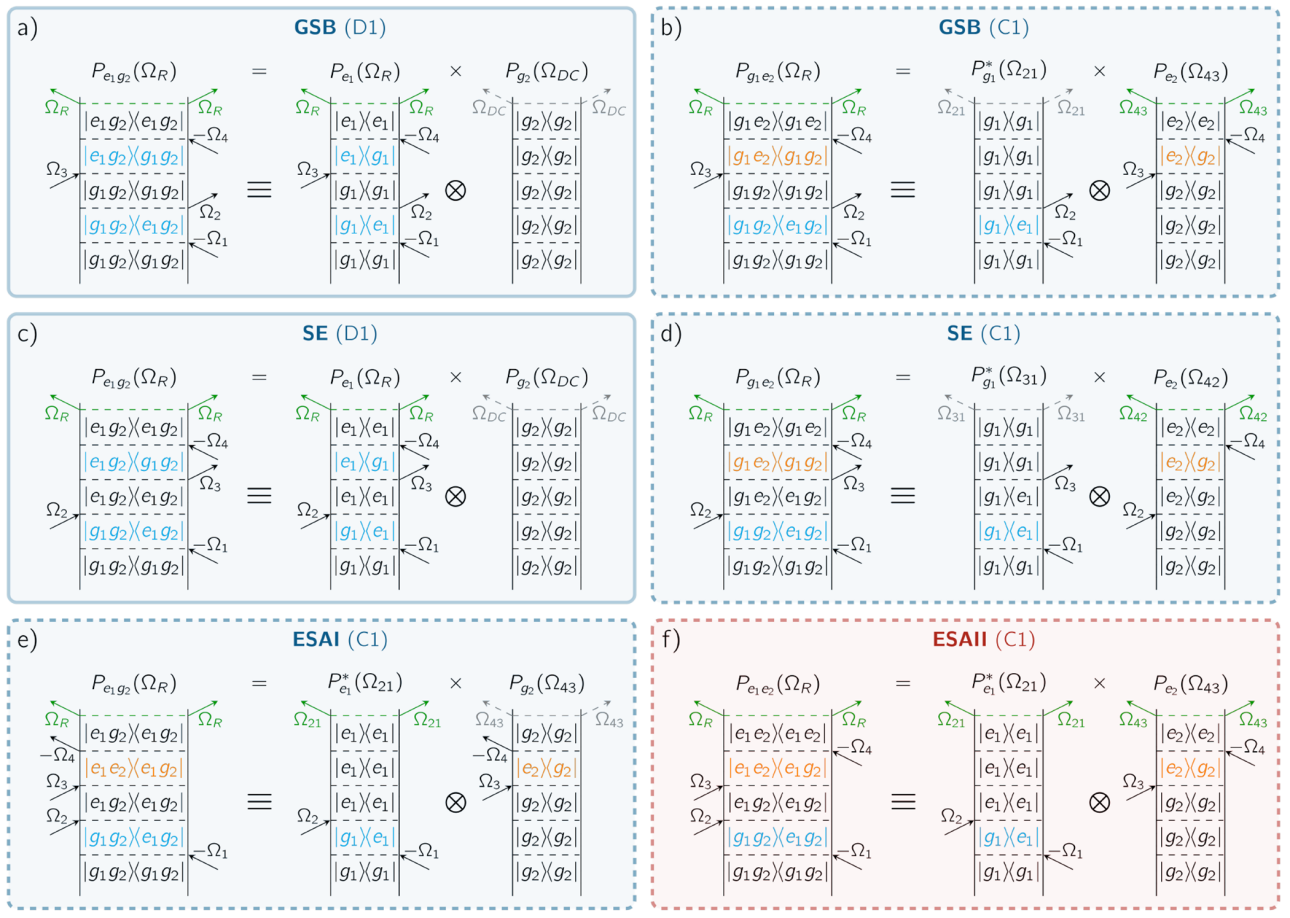
Feynman Diagrams (FDs) for the rephasing signal corresponding to (a,b) Ground-State Bleaching (GSB), (c,d) Stimulated Emission (SE), (e) Excited-State Absorption I (ESAI), and (f) Excited-State Absorption II (ESAII) pathways, along with their corresponding spectral positions as specified in Figure 2f. In each panel, the two- (2P-FD) and one-particle (1P-FD) representations of FD are depicted respectively on the left and right. At the top of each FD is reported the population, modulated at frequency Ω_*S*_, at the origin of the incoherent signal. FDs can be further distinguished in (a,c) self-population pathways (solid contour), where the same chromophore interacts with all four pulses, and (b,d–f) cross-population pathways (dashed contour), where each chromophore interacts with a different pair of pulses. Each FD can contribute with either a positive (red panel) or a negative (blue panel) sign to the signal.

The 2P-FDs can be differentiated depending on their final two-particle population. Populations *P*_*e*_1_*g*_2__ and *P*_*g*_1_*e*_2__ originate from GSB (Figure 3a,b) and SE (Figure 3c,d), which appears as both diagonal peaks and cross-peaks, and from ESAI (Figure 3e), only contributing to cross-peaks. All of these pathways are associated with spectral features of negative sign (Figure 2a,b). On the contrary, *P*_*e*_1_*e*_2__ is formed through ESAII pathways (Figure 3f), contributing with positive cross-peaks (Figure 2c).

By decomposing each 2P-FD into the product of two 1P-FDs, we can track the pathway followed by each chromophore individually. From this perspective, the pathways can be distinguished into two categories: self-population pathways (Figure 3a,c), which involve the interaction of one chromophore with all four laser pulses, and cross-population pathways (Figure 3b,d–f), in which each chromophore interacts with a different pair of pulses. This classification is introduced in analogy to that of self- and cross-polarization pathways in C-2DES, proposed by Yang and Fleming.^41^ For the considered system, self-population pathways correspond to diagonal peaks (D1 and D2), while cross-population pathways contribute as cross-peaks (C1 and C2). It follows that, at *T*_*d*_ = 0 fs, negative cross-population pathways of GSB, SE, and ESAI exactly cancel the positive cross-population contributions of ESAII. Therefore, only diagonal peaks associated with GSB and SE self-population pathways appear in the total spectrum. Indeed, these are the only pathways available for a single chromophore to generate a population modulated at the rephasing frequency Ω_*R*_ in eq 5.

However, the sum of the different spectral contributions at *T*_*d*_ = 0 fs is not what is experimentally observed, since the situation may change when taking into account the dynamics during the detection time *T*_*d*_. Starting from the end of the fourth pulse, we introduce a simple kinetic model that accounts for the relaxation processes active during signal emission. In the following, we focus the discussion on the EEA process, while considerations about the inclusion of the EET are drawn in the Supporting Information. As depicted in Figure 1c, we consider the exciton recombination at rate α_*n*_ and exciton–exciton annihilation at rates β_*n*_ and γ_12_, corresponding, respectively, to the loss of one and two excitons in the process. For simplicity, the rates of these processes are assumed to be time-independent, resulting in the following kinetic scheme for the two-particle populations:
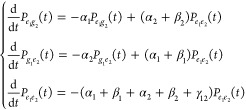
6As outlined in the Supporting Information, by solving the kinetic scheme, the time-integrated signal can be expressed in terms of two-particle populations at *T*_*d*_ = 0 fs as:
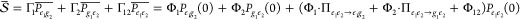
7where  and  are the quantum yields of the one-exciton states,  is the quantum yield of the two-exciton state, while Π_*e*_1_*e*_2_→*e*_1_*g*_2__ =  and Π_*e*_1_*e*_2_→*g*_1_*e*_1__ =  are the probabilities that the two-exciton state converts to one or the other one-exciton state during the detection time. In eq 7, the first and second terms are responsible for the negative contributions to the spectra (Figure 2a,b), while the third term is responsible for the positive contributions (Figure 2c). Notice that, according to their spectrum at *T*_*d*_ = 0 fs (Figure 2a–c), all of these terms give rise to cross-peaks associated with cross-population pathways (Figure 3b,d–f).

In Figure 4 are shown the time-integrated spectra for various rates α_*n*_, β_*n*_, and γ_12_, leading to different cross-peak amplitudes. When exciton recombination is faster than EEA (α_*n*_ ≫ β_*n*_, γ_12_), cross-peaks do not appear and the spectrum only reflects the contribution of individual chromophores (Figure 4a). Instead, cross-peaks start to arise when EEA competes with exciton recombination, as exemplified by the limiting cases of the net loss of one exciton (β_*n*_ ≫ α_*n*_, γ_12_) and two excitons (γ_12_ ≫ α_*n*_, β_*n*_) in Figure 4b,c, respectively. The cross-peak amplitude is determined by the balance between the positive and negative contributions from the two-particle populations in eq 7. An analysis of the peak amplitudes as a function of the different relaxation rates is reported in the Supporting Information. Therefore, in the two-particle representation, the appearance of cross-peaks at early waiting times arises from the imperfect cancellation of different pathways, as previously discussed in terms of the reduced contribution of ESAII due to EEA.^26,28,29^

**Figure 4:**
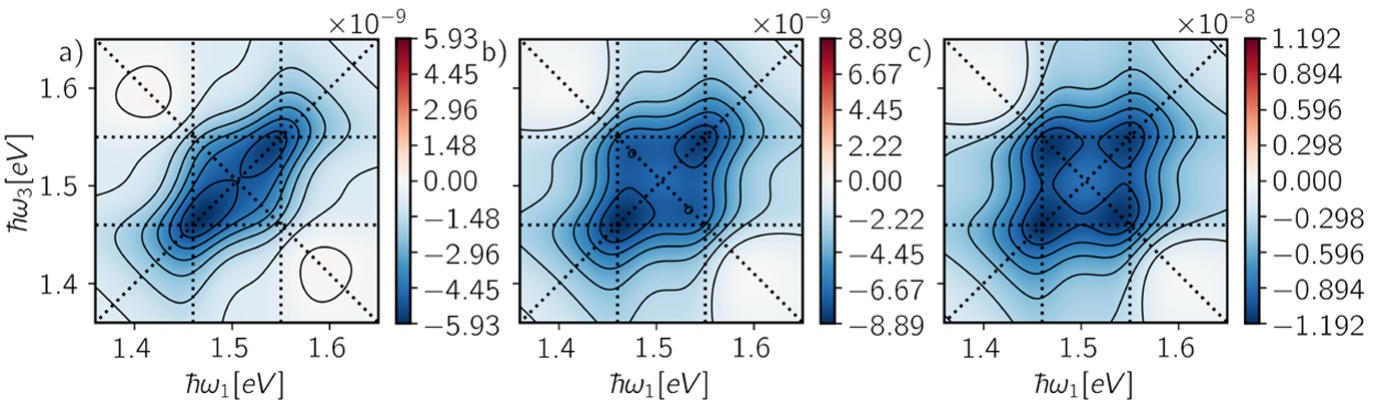
Time-integrated rephasing spectra obtained for (a) α_*n*_^–1^ = 1 ns, β_*n*_^–1^ = 1 μs, γ_12_^–1^ = 1 μs, (b) α_*n*_^–1^ = 1 ns, β_*n*_^–1^ = 1 ps, γ_12_^–1^ = 1 μs, and (c) α_*n*_^–1^ = 1 ns, β_*n*_^–1^ = 1 μs, γ_12_^–1^ = 1 ps. The emission rates of the states are set to Γ_*n*_ = 1 ns^–1^ and Γ_12_ = Γ_1_ + Γ_2_.

We now demonstrate how, for weakly interacting systems, such cross-peaks can be interpreted as incoherent mixing contributions. To this end, we derive a kinetic scheme for one-particle populations (Figure 1b) by combining the relevant kinetic equations for two-particle populations (eq 6) to obtain:
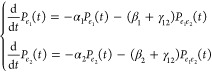
8where the two-exciton population *P*_*e*_1_*e*_2__(*t*) explicitly appears. Because at *T*_*d*_ = 0 fs the total spectrum corresponds to the sum of independent molecular responses, we employ the solution for *P*_*e*_1_*e*_2__(*t*) of eq 6 with the initial condition *P*_*e*_1_*e*_2__(0) = *P*_*e*_1__(0) × *P*_*e*_2__(0). As detailed in the Supporting Information, under this assumption, the time-integrated signal can be written in terms of the one-particle populations at *T*_*d*_ = 0 fs as follows:
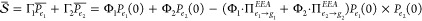
9where Π_*e*_1_→*g*_1__^EEA^ =  and Π_*e*_2_→*g*_2__^EEA^ =  are the probabilities to relax from the excited- to the ground-state of each molecule through EEA during the detection time. In this alternative decomposition of the signal, the first and second terms correspond to diagonal contributions associated with the response of individual chromophores (Figure 2d,e), while cross-peaks are generated by a third term featuring the product of one-particle populations. Notice the correspondence between the terms in the one-particle representation of the signal (eq 9) and those in the two classes of FDs (Figure 3). The first and second terms correspond to the one-particle signals , modulated at Ω_*R*_ = −Ω_1_ + Ω_2_ + Ω_3_ – Ω_4_, which originate from self-population pathways (Figure 3a,c). In the absence of EEA (β_*n*_, γ_12_ = 0), these are the only contributions appearing in the spectra, consistent with the condition of independent chromophores (Figure 4a). Instead, the third term is responsible for the contribution of incoherent mixing and corresponds to all cross-population pathways (Figure 3b,d–f). Only when EEA is active (β_*n*_, γ_12_ ≠ 0), this term leads to the appearance of negative cross-peaks in the total spectrum (Figure 4b,c). Indeed, cross-population pathways arise from the product of two 1P-FDs, in which each pair of pulses interacts with different chromophores (Figure 3b,d–f). These one-particle signals , originating from two light–matter interactions, correspond to linear signals modulated at frequency Ω_*ij*_ = Ω_*i*_ – Ω_*j*_. As a result, the product of two linear signals can also be modulated at the same frequency as the rephasing, i.e.,  is modulated at Ω_21_ – Ω_43_ = Ω_*R*_. Therefore, in this picture, the emergence of cross-peaks is due to the incoherent mixing of linear signals during the detection time.^31,32^ In this respect, the term “incoherent mixing” may be deceptive. Indeed, the mixing signal inherits and preserves the phase combination of the fourth-order interaction sequence, and for this reason, it is extracted together with the nonlinear response.

To summarize, we have demonstrated that, in the weak interaction limit, cross-peaks at early waiting times and the phenomenon of incoherent mixing correspond to alternative pictures, i.e., two- and one-particle representations, of the same underlying physical dynamics.

The analysis presented above highlights several points deserving explicit discussion. First, we notice that cross-population pathways are generated by all the processes, i.e., GSB, SE, and ESA-type pathways, and therefore, they can contribute to the spectrum with both positive and negative signs. This clarifies that the incoherent mixing contribution should not be identified exclusively with the ESAII pathways. Indeed, when annihilation is efficient and the spectrum is “ESA-free”,^29^ the GSB contribution can still be determined in part by incoherent mixing. As a consequence, no precise phase relationship is expected between the “true” nonlinear signal and incoherent mixing contributions, contrary to what has been theoretically proposed in ref (33). In fact, while in the specific model analyzed above incoherent mixing contributes with negative spectral features, it can also contribute with positive signs when the system has a second excited-state with a high emission rate. This extension of the model is explicitly considered in the Supporting Information.

Toward an unambiguous definition of incoherent mixing, it is worth emphasizing that one- and two-particle representations are equivalent only for weakly interacting chromophores. In this context, the interpretation of cross-peaks as the nonlinear signal of the composite system or as the incoherent mixing of the response of its subparts is a matter of representation dictated by the choice of what system is of interest. Whereas in the one-particle representation, the focus is on the single chromophore and cross-peaks arise from the spurious interaction with another system, the two-particle representation supports the dimeric nature of the system, even when the interaction is weak, and cross-peaks are part of the nonlinear response of the system as a whole. In the literature about A-2DES, the former view has been traditionally adopted to interpret the response of extended solid-state systems,^31,42^ while the latter has been privileged for analyzing the response of small molecular aggregates.^26−28^

Beyond the weak-coupling regime, energy splitting and dipole redistribution related to excitonic delocalization on the two chromophores must be considered. In this case, Feynman diagrams contributing to cross-peak positions (Figure 3b,d–f) no longer represent the product of one-particle signals. As a result, they do not generate incoherent mixing but rather become expressions of the nonlinear response of the molecular dimer.^26−28^

Therefore, while incoherent mixing of one-particle signals can be always recast as the net contribution of fourth-order pathways in the two-particle picture, the factorization of nonlinear pathways into the product of one-particle signals does not hold in general. This leads to the central issue of how to identify cross-peaks representing incoherent mixing. We remark the two conditions necessary to derive the one-particle representation of the signal (eq 9), namely, weak coupling between chromophores and the time scale separation between the dynamics during the delay times and the slower mixing process during the detection time. The latter condition points out that time-gating strategies^26,28,30^ can be used to reduce the contribution of incoherent mixing to the spectrum. Indeed, as reported in the Supporting Information, the time-gated signal shows how the term related to incoherent mixing grows as the integration window increases.

Recognizing the presence of incoherent mixing is especially important because the associated spectral features may hide the relevant spectral dynamics. Since in the weak coupling regime, the environments of the two chromophores can be considered as independent, cross-population pathways do not have rephasing capability.^41^ As a result, incoherent mixing contributions are not diagonally elongated and their line shape is not expected to undergo significant changes along the waiting time *T*_2_.^31^ On the one hand, this may have the detrimental effect of hiding spectral diffusion of nearby nonlinear features. On the other hand, a careful analysis of the cross-peak line shape can clarify whether they are related to incoherent mixing or excitonic delocalization. Nevertheless, the presence of incoherent mixing can be informative of the interaction network at play in the system. Indeed, it has been shown that dynamics-induced nonlinearities can be exploited to study the long-range transport mechanism in photovoltaic devices using a pump–probe setting.^42−44^

A further point deserving attention is the generality of the incoherent mixing mechanism. Indeed, just as the mixing of linear signals can enter in the fourth-order response, higher-order contributions may appear in the linear signal, e.g.,  is modulated at Ω_*R*_+Ω_43_ = Ω_21_, as recently observed in ref (32), or the mixing between fourth-order and linear signals may contribute to sixth-order response.^45^ Hence, the incoherent mixing of contributions from different orders is intrinsic to A-2DES and should always be considered in spectral assignments and simulations.

In this regard, we point out that the one-particle representation of the signal provides a numerically efficient way to simulate the effect of incoherent mixing on the action response of extended systems. In fact, the number of one-particle populations scales linearly, 2*N*, with the number of chromophores *N*, compared to the exponential scaling of the entire combinatorial space, 2^*N*^. Therefore, it is possible to simulate the response of individual subunits and then combine them by using a kinetic scheme for one-particle populations. Notice that a similar kinetic scheme has been derived assuming the independence of the excited-state population of each chromophore at every time, *P*_*e*_1_*e*_2__(*t*) = *P*_*e*_1__(*t*) × *P*_*e*_2__(*t*), obtaining proper nonlinear population dynamics.^46^ In the continuum limit, the product between populations can be replaced by a quadratic term of the form *P*(**r**, *t*)^2^, as originally used to define the concept of incoherent mixing.^31^ In light of these considerations, the analysis can be generalized to supramolecular complexes, e.g., the LH2 complex, composed of weakly coupled domains, e.g., B800 and B850 rings, interacting only during the detection time. In this case, both the nonlinear response of each domain and the incoherent mixing between different domains can contribute to the signal, eventually overlapping in the spectrum.

In conclusion, through the use of one- and two-particle representations, we have clarified the nature and role of incoherent mixing in A-2DES spectra of weakly interacting systems. Overlooking the experimental feasibility of distinguishing between these observables, one- and two-particle populations have been employed as interpretative tools to identify the dynamical pathways stemming from different orders in the light–matter interaction. Although giving equivalent results in the limit of weakly interacting systems, the two representations provide different perspectives for interpreting the emergence of spectral features. Notably, the one-particle representation makes evident the distinct nature of self-population and cross-population pathways, thus elucidating the contribution of incoherent mixing in the action-detected spectra.

## Supporting Information Available

Nonperturbative simulation procedure and parameters, population observables, response functions and Feynman diagrams, kinetic schemes for one- and two-particle populations during the detection time, detailed analysis for the chromophoric pair of two- and three-level systems (PDF)
Transparent Peer Review report available (PDF)

## References

[ref1] Fuller F. D.; Ogilvie J. P. Experimental implementations of two-dimensional Fourier transform electronic spectroscopy. Annu. Rev. Phys. Chem. 2015, 66, 667–690. 10.1146/annurev-physchem-040513-103623. 25664841

[ref2] Collini E. 2D electronic spectroscopic techniques for quantum technology Applications. J. Phys. Chem. C 2021, 125, 13096–13108. 10.1021/acs.jpcc.1c02693. PMC8282191 34276867

[ref3] Biswas S.; Kim J.; Zhang X.; Scholes G. D. Coherent two-dimensional and broadband electronic spectroscopies. Chem. Rev. 2022, 122, 4257–4321. 10.1021/acs.chemrev.1c00623. 35037757

[ref4] Tiwari V. Multidimensional electronic spectroscopy in high-definition—Combining spectral, temporal, and spatial resolutions. J. Chem. Phys. 2021, 154, 230901 10.1063/5.0052234. 34241275

[ref5] Karki K. J.; Ciappina M. F. Advances in nonlinear spectroscopy using phase modulated light fields: prospective applications in perturbative and non-perturbative regimes. Adv. Phys.: X 2022, 7, 2090856 10.1080/23746149.2022.2090856.

[ref6] Tian P.; Keusters D.; Suzaki Y.; Warren W. S. Femtosecond phase-coherent two-dimensional spectroscopy. Science 2003, 300, 1553–1555. 10.1126/science.1083433. 12791987

[ref7] Tan H.-S. Theory and phase-cycling scheme selection principles of collinear phase coherent multi-dimensional optical spectroscopy. J. Chem. Phys. 2008, 129, 124501 10.1063/1.2978381. 19045030

[ref8] Tekavec P. F.; Lott G. A.; Marcus A. H. Fluorescence-detected two-dimensional electronic coherence spectroscopy by acousto-optic phase modulation. J. Chem. Phys. 2007, 127, 214307 10.1063/1.2800560. 18067357

[ref9] Lott G. A.; Perdomo-Ortiz A.; Utterback J. K.; Widom J. R.; Aspuru-Guzik A.; Marcus A. H. Conformation of self-assembled porphyrin dimers in liposome vesicles by phase-modulation 2D fluorescence spectroscopy. Proc. Natl. Acad. Sci. U.S.A. 2011, 108, 16521–16526. 10.1073/pnas.1017308108. 21940499 PMC3189026

[ref10] Tiwari V.; Matutes Y. A.; Konar A.; Yu Z.; Ptaszek M.; Bocian D. F.; Holten D.; Kirmaier C.; Ogilvie J. P. Strongly coupled bacteriochlorin dyad studied using phase-modulated fluorescence-detected two-dimensional electronic spectroscopy. Opt. Express 2018, 26, 22327–22341. 10.1364/OE.26.022327. 30130927

[ref11] Karki K. J.; Chen J.; Sakurai A.; Shi Q.; Gardiner A. T.; Kühn O.; Cogdell R. J.; Pullerits T. Before Förster. Initial excitation in photosynthetic light harvesting. Chem. Sci. 2019, 10, 7923–7928. 10.1039/C9SC01888C. 31673317 PMC6788518

[ref12] Nardin G.; Autry T. M.; Silverman K. L.; Cundiff S. T. Multidimensional coherent photocurrent spectroscopy of a semiconductor nanostructure. Opt. Express 2013, 21, 28617–28627. 10.1364/OE.21.028617. 24514373

[ref13] Karki K. J.; Widom J. R.; Seibt J.; Moody I.; Lonergan M. C.; Pullerits T.; Marcus A. H. Coherent two-dimensional photocurrent spectroscopy in a PbS quantum dot photocell. Nat. Commun. 2014, 5, 5869 10.1038/ncomms6869. 25519819

[ref14] Bolzonello L.; Bernal-Texca F.; Gerling L. G.; Ockova J.; Collini E.; Martorell J.; van Hulst N. F. Photocurrent-detected 2D electronic spectroscopy reveals ultrafast hole transfer in operating PM6/Y6 organic solar cells. J. Phys. Chem. Lett. 2021, 12, 3983–3988. 10.1021/acs.jpclett.1c00822. 33877838 PMC8154857

[ref15] Roeding S.; Brixner T. Coherent two-dimensional electronic mass spectrometry. Nat. Commun. 2018, 9, 2519 10.1038/s41467-018-04927-w. 29955042 PMC6023891

[ref16] Uhl D.; Bangert U.; Bruder L.; Stienkemeier F. Coherent optical 2D photoelectron spectroscopy. Optica 2021, 8, 1316–1324. 10.1364/OPTICA.434853.

[ref17] Bakulin A. A.; Silva C.; Vella E. Ultrafast spectroscopy with photocurrent detection: watching excitonic optoelectronic systems at work. J. Phys. Chem. Lett. 2016, 7, 250–258. 10.1021/acs.jpclett.5b01955. 26711855 PMC4819534

[ref18] Wang C.; Cai J.; Liu X.; Chen C.; Chen X.; Karki K. J. In operando quantification of single and multiphoton photocurrents in GaP and InGaN photodetectors with phase-modulated femtosecond light pulses. ACS Photonics 2023, 10, 1119–1125. 10.1021/acsphotonics.2c01851.

[ref19] Tiwari V.; Matutes Y. A.; Gardiner A. T.; Jansen T. L. C.; Cogdell R. J.; Ogilvie J. P. Spatially-resolved fluorescence-detected two-dimensional electronic spectroscopy probes varying excitonic structure in photosynthetic bacteria. Nat. Commun. 2018, 9, 4219 10.1038/s41467-018-06619-x. 30310070 PMC6181999

[ref20] Bangert U.; Stienkemeier F.; Bruder L. High-resolution two-dimensional electronic spectroscopy reveals the homogeneous line profile of chromophores solvated in nanoclusters. Nat. Commun. 2022, 13, 3350 10.1038/s41467-022-31021-z. 35688839 PMC9187667

[ref21] Fersch D.; Malý P.; Rühe J.; Lisinetskii V.; Hensen M.; Würthner F.; Brixner T. Single-molecule ultrafast fluorescence-detected pump-probe microscopy. J. Phys. Chem. Lett. 2023, 14, 4923–4932. 10.1021/acs.jpclett.3c00839. 37207316

[ref22] Perdomo-Ortiz A.; Widom J. R.; Lott G. A.; Aspuru-Guzik A.; Marcus A. H. Conformation and electronic population transfer in membrane-supported self-assembled porphyrin dimers by 2D fluorescence spectroscopy. J. Phys. Chem. B 2012, 116, 10757–10770. 10.1021/jp305916x. 22882118

[ref23] Kjellberg P.; Brüggemann B.; Pullerits T. o. Two-dimensional electronic spectroscopy of an excitonically coupled dimer. Phys. Rev. B 2006, 74, 024303 10.1103/PhysRevB.74.024303.

[ref24] Cipolloni M.; Fresch B.; Occhiuto I.; Rukin P.; Komarova K. G.; Cecconello A.; Willner I.; Levine R. D.; Remacle F.; Collini E. Coherent electronic and nuclear dynamics in a rhodamine heterodimer–DNA supramolecular complex. Phys. Chem. Chem. Phys. 2017, 19, 23043–23051. 10.1039/C7CP01334E. 28817145

[ref25] Malý P.; Lüttig J.; Mueller S.; Schreck M. H.; Lambert C.; Brixner T. Coherently and fluorescence-detected two-dimensional electronic spectroscopy: direct comparison on squaraine dimers. Phys. Chem. Chem. Phys. 2020, 22, 21222–21237. 10.1039/D0CP03218B. 32930273

[ref26] Malý P.; Mančal T. Signatures of exciton delocalization and exciton-exciton annihilation in fluorescence-detected two-dimensional coherent spectroscopy. J. Phys. Chem. Lett. 2018, 9, 5654–5659. 10.1021/acs.jpclett.8b02271. 30188728

[ref27] Schröter M.; Pullerits T.; Kühn O. Using fluorescence detected two-dimensional spectroscopy to investigate initial exciton delocalization between coupled chromophores. J. Chem. Phys. 2018, 149, 114107 10.1063/1.5046645. 30243281

[ref28] Kunsel T.; Tiwari V.; Matutes Y. A.; Gardiner A. T.; Cogdell R. J.; Ogilvie J. P.; Jansen T. L. C. Simulating fluorescence-detected two-simensional electronic spectroscopy of multichromophoric systems. J. Phys. Chem. B 2019, 123, 394–406. 10.1021/acs.jpcb.8b10176. 30543283 PMC6345114

[ref29] Kühn O.; Mančal T.; Pullerits T. Interpreting fluorescence detected two-dimensional electronic spectroscopy. J. Phys. Chem. Lett. 2020, 11, 838–842. 10.1021/acs.jpclett.9b03851. 32024369

[ref30] Bruschi M.; Gallina F.; Fresch B. Simulating action-2D electronic spectroscopy of quantum dots: insights on the exciton and biexciton interplay from detection-mode and time-gating. Phys. Chem. Chem. Phys. 2022, 24, 27645–27659. 10.1039/D2CP04270C. 36349664

[ref31] Grégoire P.; Srimath Kandada A. R.; Vella E.; Tao C.; Leonelli R.; Silva C. Incoherent population mixing contributions to phase-modulation two-dimensional coherent excitation spectra. J. Chem. Phys. 2017, 147, 114201 10.1063/1.4994987. 28938824

[ref32] Bargigia I.; Gutiérrez-Meza E.; Valverde-Chávez D. A.; Marques S. R.; Srimath Kandada A. R.; Silva C. Identifying incoherent mixing effects in the coherent two-dimensional photocurrent excitation spectra of semiconductors. J. Chem. Phys. 2022, 157, 204202 10.1063/5.0121635. 36456239

[ref33] Kalaee A. A. S.; Damtie F.; Karki K. J. Differentiation of true nonlinear and incoherent mixing of linear signals in action-detected 2D spectroscopy. J. Phys. Chem. A 2019, 123, 4119–4124. 10.1021/acs.jpca.9b01129. 30998014

[ref34] Mukamel S. Communication: The origin of many-particle signals in nonlinear optical spectroscopy of non-interacting particles. J. Chem. Phys. 2016, 145, 041102 10.1063/1.4960049. 27475341

[ref35] Damtie F. A.; Wacker A.; Pullerits T. o.; Karki K. J. Two-dimensional action spectroscopy of excitonic systems: Explicit simulation using a phase-modulation technique. Phys. Rev. A 2017, 96, 053830 10.1103/PhysRevA.96.053830.

[ref36] Anda A.; Cole J. H. Two-dimensional spectroscopy beyond the perturbative limit: The influence of finite pulses and detection modes. J. Chem. Phys. 2021, 154, 114113 10.1063/5.0038550. 33752354

[ref37] van Grondelle R. Excitation energy transfer, trapping and annihilation in photosynthetic systems. Biochim. Biophys. Acta - Bioenerg. 1985, 811, 147–195. 10.1016/0304-4173(85)90017-5.

[ref38] van Amerongen H.; van Grondelle R.; Valkunas L. Photosynthetic excitons; World Scientific: Singapore, 2000.

[ref39] May V.; Kühn O. Charge and energy transfer dynamics in molecular systems; Wiley-VCH: Weinheim, 2011.

[ref40] Mukamel S. Principles of nonlinear optical spectroscopy; Oxford University Press: Oxford, 1995.

[ref41] Yang M.; Fleming G. R. Third-order nonlinear optical response of energy transfer systems. J. Chem. Phys. 1999, 111, 27–39. 10.1063/1.479359.

[ref42] McNamee M. G.; Ouyang Z.; Yan L.; Gan Z.; Zhou N.; Williams O. F.; You W.; Moran A. M. Uncovering transport mechanisms in perovskite materials and devices with recombination-induced action spectroscopies. J. Phys. Chem. C 2023, 127, 2782–2791. 10.1021/acs.jpcc.2c08851.

[ref43] Zhou N.; Ouyang Z.; Hu J.; Williams O. F.; Yan L.; You W.; Moran A. M. Distinguishing energy- and charge-transfer processes in layered perovskite quantum wells with two-dimensional action spectroscopies. J. Phys. Chem. Lett. 2020, 11, 4570–4577. 10.1021/acs.jpclett.0c00844. 32428411

[ref44] Ouyang Z.; Zhou N.; McNamee M. G.; Yan L.; Williams O. F.; You W.; Moran A. M. Multidimensional time-of-flight spectroscopy. J. Chem. Phys. 2021, 154, 220901 10.1063/5.0047382. 34241190

[ref45] Malý P.; Mueller S.; Lüttig J.; Lambert C.; Brixner T. Signatures of exciton dynamics and interaction in coherently and fluorescence-detected four- and six-wave-mixing two-dimensional electronic spectroscopy. J. Chem. Phys. 2020, 153, 144204 10.1063/5.0022743. 33086839

[ref46] May V. Kinetic theory of exciton-exciton annihilation. J. Chem. Phys. 2014, 140, 054103 10.1063/1.4863259. 24511918

