# Supplementary material for: Unifying Nonlinear Response and Incoherent Mixing in Action-2D Electronic Spectroscopy
Source: J Phys Chem Lett. 2023 Jul 25;14(30):6872–9. doi: 10.1021/acs.jpclett.3c01670 (PMC10405272; doi:10.1021/acs.jpclett.3c01670)
Supplement: Supplementary file 1 — jz3c01670_si_001.pdf [file jz3c01670_si_001.pdf]

Supporting Information
Unifying Non-Linear Response and Incoherent Mixing in Action-2D Electronic Spectroscopy

Matteo Bruschi[1], Luca Bolzonello[2], Federico Gallina[1], and Barbara Fresch[1,3]

[1]Dipartimento di Scienze Chimiche, Università degli Studi di Padova, via Marzolo 1, Padua 35131, Italy
[2]ICFO - Institut de Ciencies Fotoniques, The Barcelona Institute of Science and Technology, Castelldefels, Barcelona, 08860 Spain
[3]Padua Quantum Technologies Research Center, Università degli Studi di Padova, Italy

Contents

1 Non-Perturbative Simulation **S2**
1.1 Phase-Modulation Scheme . . . S2
1.2 System Hamiltonian . . . S2
1.3 Light-Matter Hamiltonian . . . S2
1.4 Lindblad Quantum Master Equation . . . S3

2 Population Observables **S3**
2.1 Two-Particle Populations . . . S3
2.2 One-Particle Populations . . . S3
2.3 Relations between One- and Two-Particle Populations . . . S4

3 Response Theory and Feynman Diagrams **S4**
3.1 Response Functions . . . S4
3.1.1 Second-Order Response Functions . . . S4
3.1.2 Fourth-Order Response Functions . . . S5
3.2 Feynman Diagrams in One- and Two-Particle Representations . . . S5

4 Kinetic Scheme for Populations and Incoherent Signal **S5**
4.1 Two-Particle Populations . . . S6
4.2 One-Particle Populations . . . S8

5 Generalized Kinetic Scheme **S9**

6 Chromophoric Pair of Two-Level Systems **S10**

7 Chromophoric Pair of Three-Level Systems **S12**

1 Non-Perturbative Simulation

In order to simulate the optical response of A-2DES, we employ a non-perturbative treatment of the light-matter interaction, by explicitly incorporating the electric-field with its phase-modulation pattern in the Hamiltonian, as outlined in refs. [1, 2]. The dynamics of the system is simulated using the Lindblad quantum master equation for the system density matrix.

1.1 Phase-Modulation Scheme

In A-2DES, the system interacts with a train of four phase-modulated laser pulses. The electric-field of the m-th train is:

$$E^m(t) = \sum_{i=1}^{4} E_i^m(t) \tag{1}$$

with the i-th pulse described by the convolution of a Gaussian envelope and an oscillating function:

$$E_i^m(t) = E_i^0 \exp\left\{ -\frac{(t - \mathcal{T}_i)^2}{2\sigma_i^2} \right\} \cos[\omega_i(t - \mathcal{T}_i) + \Phi(\Omega_i)]. \tag{2}$$

where E_i^0 is the electric-field amplitude, σ_i is the pulse duration, and ω_i is the carrier-frequency. The first pulse is centered at time $T_0 = t_1$, while the following pulses are separated from the previous one by delay-times $T_1 = t_2 - t_1$, $T_2 = t_3 - t_2$, and $T_3 = t_4 - t_3$. For convenience, we make use of a cumulative delay-time: $\mathcal{T}_i = \sum_{j=0}^{i-1} T_j$. In a phase-modulation scheme, the phase of each pulse is linearly varied from a train to the following as $\Phi(\Omega_i) = 2\pi\Omega_i mT$, where Ω_i is the phase-modulation frequency, T is the inter-train delay-time and m is an integer which is incremented from 0 to $M - 1$.

In the simulations, the following parameters are used: σ_i = 5 fs, $\hbar\omega_i$ = 1.50 eV, Ω_1 = 0 Hz, Ω_2 = 250 Hz, Ω_3 = 600 Hz, Ω_4 = 1000 Hz, $M = 60$ and $T = 1/3000$ s. The delay-times T_1 and T_3 are scanned from 0 to 300 fs in steps of 10 fs, while the waiting-time is kept fixed at $T_2 = 0$ fs. The signal is acquired in the rotating frame at angular frequency $\omega_{RF} = 2.28$ [rad]/fs. In order to smooth the spectra, the signal is zero-padded in the time domain.

1.2 System Hamiltonian

The chromophoric pair is described by the Hamiltonian:

$$\hat{H} = \hat{H}_1 \otimes \mathbb{1}_2 + \mathbb{1}_1 \otimes \hat{H}_2 + \hat{V}_{12} \tag{3}$$

where $\hat{H}_n = \epsilon_n |e_n\rangle\langle e_n|$ and $\mathbb{1}_n = |g_n\rangle\langle g_n| + |e_n\rangle\langle e_n|$ are respectively the Hamiltonian and the identity operator of the n-th chromophore, while $\hat{V}_{12}$ is the excitonic coupling between the two chromophores. In the simulations, the excitation energy of the two chromophores are respectively ϵ_1 = 1.55 eV and ϵ_2 = 1.46 eV, while the Hamiltonian term of the excitonic coupling is assumed to be vanishing.

1.3 Light-Matter Hamiltonian

In the dipole approximation, the light-matter interaction is described by the Hamiltonian:

$$\hat{H}'(t) = -\hat{\mu} \cdot E(t) \tag{4}$$

where $E(t)$ is the electric-field, defined in Eqs. 1-2, and:

$$\hat{\mu} = \hat{\mu}_1 \otimes \mathbb{1}_2 + \mathbb{1}_1 \otimes \hat{\mu}_2 \tag{5}$$

is the dipole moment operator of the chromophoric pair. For the n-th chromophore, the dipole moment operator is defined as:

$$\hat{\mu}_n = \mu_n\big(|g_n\rangle\langle e_n| + |e_n\rangle\langle g_n| \big) \tag{6}$$

where μ_n is its transition dipole moment. In the simulations, the dipole moments of the two chromophores are assumed to be parallel to the electric-field, and the light-matter coupling strength is $\mu_n E_i^0$ = 3 meV.

1.4 Lindblad Quantum Master Equation

The dynamics of the system is obtained by solving the Lindblad quantum master equation:

$$\frac{d}{dt}\rho(t) = -\frac{i}{\hbar}\left[\hat{H}_S, \rho(t)\right] - \frac{i}{\hbar}\left[\hat{H}'(t), \rho(t)\right] + \sum_k \frac{\gamma_k}{\hbar}\left(\hat{L}_k\rho(t)\hat{L}_k^\dagger - \frac{1}{2}\left\{\hat{L}_k^\dagger\hat{L}_k, \rho(t)\right\}\right) \tag{7}$$

where $\rho(t)$ is the system density matrix, $\hat{H}$ is the system Hamiltonian (Eq. 3) and $\hat{H}'(t)$ is the light-matter interaction Hamiltonian (Eq. 4). The presence of dissipation and decoherence processes, as a consequence of interaction with the environment, is introduced by defining the Lindblad operators $\hat{L}_k$ with associated rates $\gamma_k/\hbar$.

In the simulations, it is assumed that the chromophoric pair is initially in the collective ground-state, $\rho(0) = |g_1g_2\rangle\langle g_1g_2|$. During the coherent excitation induced by the laser pulses, we only account for decoherence processes as described by the Lindblad operators:

$$\begin{aligned}
\hat{L}_1 &= |g_1\rangle\langle g_1| \otimes \mathbb{1}_2 = |g_1g_2\rangle\langle g_1g_2| + |g_1e_2\rangle\langle g_1e_2| \\
\hat{L}_2 &= |e_1\rangle\langle e_1| \otimes \mathbb{1}_2 = |e_1g_2\rangle\langle e_1g_2| + |e_1e_2\rangle\langle e_1e_2| \\
\hat{L}_3 &= \mathbb{1}_1 \otimes |g_2\rangle\langle g_2| = |g_1g_2\rangle\langle g_1g_2| + |e_1g_2\rangle\langle e_1g_2| \\
\hat{L}_4 &= \mathbb{1}_1 \otimes |e_2\rangle\langle e_2| = |g_1e_2\rangle\langle g_1e_2| + |e_1e_2\rangle\langle e_1e_2|
\end{aligned} \tag{8}$$

with associated rates set all equal $\gamma_k/h = 1/100$ fs. This choice for the Lindblad operators is equivalent to assume that each chromophore has an independent environment.

2 Population Observables

The non-perturbative simulation results in the density matrix of the system $\rho(t)$, after the interaction with the train of pulses. Based on this density matrix, it is possible to extract various observables of the system. In the case of A-2DES, we are interested in excited-state populations, in particular. In the following, we formally introduce one- and two-particle populations along with the relations between them.

2.1 Two-Particle Populations

Two-particle populations can be obtained by taking the expectation values:

$$\begin{aligned}
P_{g_1g_2}(t) &= \mathrm{Tr}\{|g_1g_2\rangle\langle g_1g_2|\,\rho(t)\} \\
P_{e_1g_2}(t) &= \mathrm{Tr}\{|e_1g_2\rangle\langle e_1g_2|\,\rho(t)\} \\
P_{g_1e_2}(t) &= \mathrm{Tr}\{|g_1e_2\rangle\langle g_1e_2|\,\rho(t)\} \\
P_{e_1e_2}(t) &= \mathrm{Tr}\{|e_1e_2\rangle\langle e_1e_2|\,\rho(t)\}
\end{aligned} \tag{9}$$

which represent the probabilities that the dimer is found in a certain two-particle state.

2.2 One-Particle Populations

One-particle populations can be obtained by taking the expectation values:

$$\begin{aligned}
P_{g_1}(t) &= \mathrm{Tr}\{|g_1\rangle\langle g_1| \otimes \mathbb{1}_2\,\rho(t)\} \\
P_{e_1}(t) &= \mathrm{Tr}\{|e_1\rangle\langle e_1| \otimes \mathbb{1}_2\,\rho(t)\} \\
P_{g_2}(t) &= \mathrm{Tr}\{\mathbb{1}_1 \otimes |g_2\rangle\langle g_2|\,\rho(t)\} \\
P_{e_2}(t) &= \mathrm{Tr}\{\mathbb{1}_1 \otimes |e_2\rangle\langle e_2|\,\rho(t)\}.
\end{aligned} \tag{10}$$

which represent the probabilities that one of the two monomer is found in a certain state independently of the other.

2.3 Relations between One- and Two-Particle Populations

From Eq. 10, it results that one-particle populations are related to two-particle populations by the sum:

$$\begin{aligned} P_{g_1}(t) &= P_{g_1g_2}(t) + P_{g_1e_2}(t) \\ P_{g_2}(t) &= P_{g_1g_2}(t) + P_{e_1g_2}(t) \\ P_{e_1}(t) &= P_{e_1g_2}(t) + P_{e_1e_2}(t) \\ P_{e_2}(t) &= P_{g_1e_2}(t) + P_{e_1e_2}(t) \end{aligned} \tag{11}$$

which is equivalent to take the partial trace over the states of the other molecule. Notice that it is always possible to obtain one-particle populations from two-particle populations.

In contrast, two-particle populations cannot be expressed in terms of one-particle populations in general. However, if we make the assumption that, at the time $t = t_0$, the populations of the two chromophores are uncorrelated, then two-particle populations can be factorized as:

$$\begin{aligned} P_{g_1g_2}(t_0) &= P_{g_1}(t_0) \times P_{g_2}(t_0) \\ P_{e_1g_2}(t_0) &= P_{e_1}(t_0) \times P_{g_2}(t_0) \\ P_{g_1e_2}(t_0) &= P_{g_1}(t_0) \times P_{e_2}(t_0) \\ P_{e_1e_2}(t_0) &= P_{e_1}(t_0) \times P_{e_1}(t_0). \end{aligned} \tag{12}$$

In the main text, we consider this specific situation at detection-time $T_d = 0$ fs, after the interaction with the train of pulses. Since the two chromophores do not interact during the coherent dynamics induced by the laser pulses, the factorization of two-particle populations in terms of one-particle populations holds. Analogously, also the density matrix of the chromophoric pair can be factorized into the density matrices of the individual chromophores as $\rho(0) = \rho_1(0) \otimes \rho_2(0)$.

3 Response Theory and Feynman Diagrams

In this section, we provide the expressions for the response functions and report the complete set of Feynman Diagrams for the rephasing signal of A-2DES in the case of the weakly-interacting chromophores.

3.1 Response Functions

In formulating the response functions, we make several assumptions: the impulse limit, the time-ordering of the pulses, and the Rotating-Wave Approximation (RWA). Additionally, for simplicity, we assume the unitary evolution of the system under the Hamiltonian H (Eq. 3) between the pulses.

3.1.1 Second-Order Response Functions

For the linear signals modulated at frequency $\Omega_{ij} = \Omega_i - \Omega_j$, the second-order response functions are:

$$\mathcal{J}_1^{(n)}(t_{ij}) = +\left(\frac{i}{\hbar}\right)^2 |\mu_n|^2 e^{-i\omega_n t_{ij}} e^{+i\Phi(\Omega_{ij})} \tag{13}$$

$$\mathcal{J}_2^{(n)}(t_{ij}) = -\left(\frac{i}{\hbar}\right)^2 |\mu_n|^2 e^{-i\omega_n t_{ij}} e^{+i\Phi(\Omega_{ij})} \tag{14}$$

where the superscript index (n) represents the interaction with the n-th chromophore, with $n = 1, 2$. In the expression, μ_n is the transition dipole moment, $\omega_n = \epsilon_n/\hbar$ is the transition frequency, $\Phi(\Omega_{ij})$ is the phase associated to the pathway as a result of phase-modulation, and $t_{ij} = t_i - t_j$ is the delay-time between two pulses centered respectively at t_i and t_j.

3.1.2 Fourth-Order Response Functions

For the rephasing signal modulated at frequency $\Omega_R = -\Omega_1 + \Omega_2 + \Omega_3 - \Omega_4$, the fourth-order response functions are, for Ground-State Bleaching (GSB):

$$\mathcal{R}^{(n,n)}_{GSB}(t_{21}, t_{43}) = -\left(\frac{i}{\hbar}\right)^4 |\mu_n|^4 e^{+i\omega_n t_{21}} e^{-i\omega_n t_{43}} e^{+i\Phi(\Omega_R)} \tag{15}$$

$$\mathcal{R}^{(n,m)}_{GSB}(t_{21}, t_{43}) = -\left(\frac{i}{\hbar}\right)^4 |\mu_n|^2|\mu_m|^2 e^{+i\omega_n t_{21}} e^{-i\omega_m t_{43}} e^{+i\Phi(\Omega_R)} = \left(\mathcal{J}^{(n)}_2(t_{21})\right)^* \times \mathcal{J}^{(m)}_1(t_{43}) \tag{16}$$

for Stimulated Emission (SE):

$$\mathcal{R}^{(n,n)}_{SE}(t_{21}, t_{43}) = -\left(\frac{i}{\hbar}\right)^4 |\mu_n|^4 e^{+i\omega_n t_{21}} e^{-i\omega_n t_{43}} e^{+i\Phi(\Omega_R)} \tag{17}$$

$$\mathcal{R}^{(n,m)}_{SE}(t_{31}, t_{42}) = -\left(\frac{i}{\hbar}\right)^4 |\mu_n|^2|\mu_m|^2 e^{+i\omega_n t_{31}} e^{-i\omega_m t_{42}} e^{+i\Phi(\Omega_R)} = \left(\mathcal{J}^{(n)}_2(t_{31})\right)^* \times \mathcal{J}^{(m)}_1(t_{42}) \tag{18}$$

for Excited-State Absorption I (ESAI):

$$R^{(n,m)}_{ESAI}(t_{31}, t_{42}) = -\left(\frac{i}{\hbar}\right)^4 |\mu_n|^2|\mu_m|^2 e^{+i\omega_n t_{21}} e^{-i\omega_m t_{43}} e^{+i\Phi(\Omega_R)} = \left(\mathcal{J}^{(n)}_1(t_{21})\right)^* \times \mathcal{J}^{(m)}_2(t_{43}) \tag{19}$$

$$\mathcal{R}^{(n,m)}_{ESAI}(t_{21}, t_{43}) = -\left(\frac{i}{\hbar}\right)^4 |\mu_n|^2|\mu_m|^2 e^{+i\omega_n t_{31}} e^{-i\omega_m t_{42}} e^{+i\Phi(\Omega_R)} = \left(\mathcal{J}^{(n)}_1(t_{31})\right)^* \times \mathcal{J}^{(m)}_2(t_{42}) \tag{20}$$

and for Excited-State Absorption II (ESAII):

$$\mathcal{R}^{(n,m)}_{ESAII}(t_{31}, t_{42}) = +\left(\frac{i}{\hbar}\right)^4 |\mu_n|^2|\mu_m|^2 e^{+i\omega_n t_{21}} e^{-i\omega_m t_{43}} e^{+i\Phi(\Omega_R)} = \left(\mathcal{J}^{(n)}_2(t_{21})\right)^* \times \mathcal{J}^{(m)}_2(t_{43}) \tag{21}$$

$$\mathcal{R}^{(n,m)}_{ESAII}(t_{21}, t_{43}) = +\left(\frac{i}{\hbar}\right)^4 |\mu_n|^2|\mu_m|^2 e^{+i\omega_n t_{31}} e^{-i\omega_m t_{42}} e^{+i\Phi(\Omega_R)} = \left(\mathcal{J}^{(n)}_2(t_{31})\right)^* \times \mathcal{J}^{(m)}_2(t_{42}). \tag{22}$$

Fourth-order response functions can be differentiated into self-population pathways [labeled with (n, n)], if the interaction with the pulses only involves the n-th chromophore, and cross-population pathways [labeled with (n, m)], if the interaction involves both chromophores n and m. Notice that, in the case of cross-population pathways, the fourth-order response function can be decomposed into the product of second-order response functions of each chromophore, emphasizing how they originate from the product of linear signals.

In addition, we report that some cross-population pathways associated to SE (Eq. 18), ESAI (Eq. 20) and ESAII (Eq. 22) contributions exhibit a coherence during the waiting-time $T_2 = t_{32} = t_3 - t_2$. For the considered weakly-interacting system, these coherences are inter-site rather than inter-exciton in nature [3]. In the case of independent environments of the two chromophores, these coherences are expected to quickly dephase along T_2, but they may still contribute to the signal at short waiting-time to some extent.

3.2 Feynman Diagrams in One- and Two-Particle Representations

In the following, we report the complete set of Feynman diagrams for the rephasing signal modulated at $\Omega_R = -\Omega_1 + \Omega_2 + \Omega_3 - \Omega_4$ in the one-particle (1P-FD) and two-particle (2P-FD) representations. The various FDs can be distinguished depending on the two-particle population from which the signal is emitted, i.e., $P_{e_1 g_2}$ (Fig. S1), $P_{g_1 e_2}$ (Fig. S2), and $P_{e_1 e_2}$ (Fig. S3). In each panel of Fig. S1-S3, 2P-FDs are reported on the left while the equivalent decomposition in 1P-FDs is reported on the right.

4 Kinetic Scheme for Populations and Incoherent Signal

In this section, we present a kinetic scheme for the one- and two-particle populations during the detection-time T_d. The initial populations can be obtained either from non-perturbative simulations (Sec. 1) or from

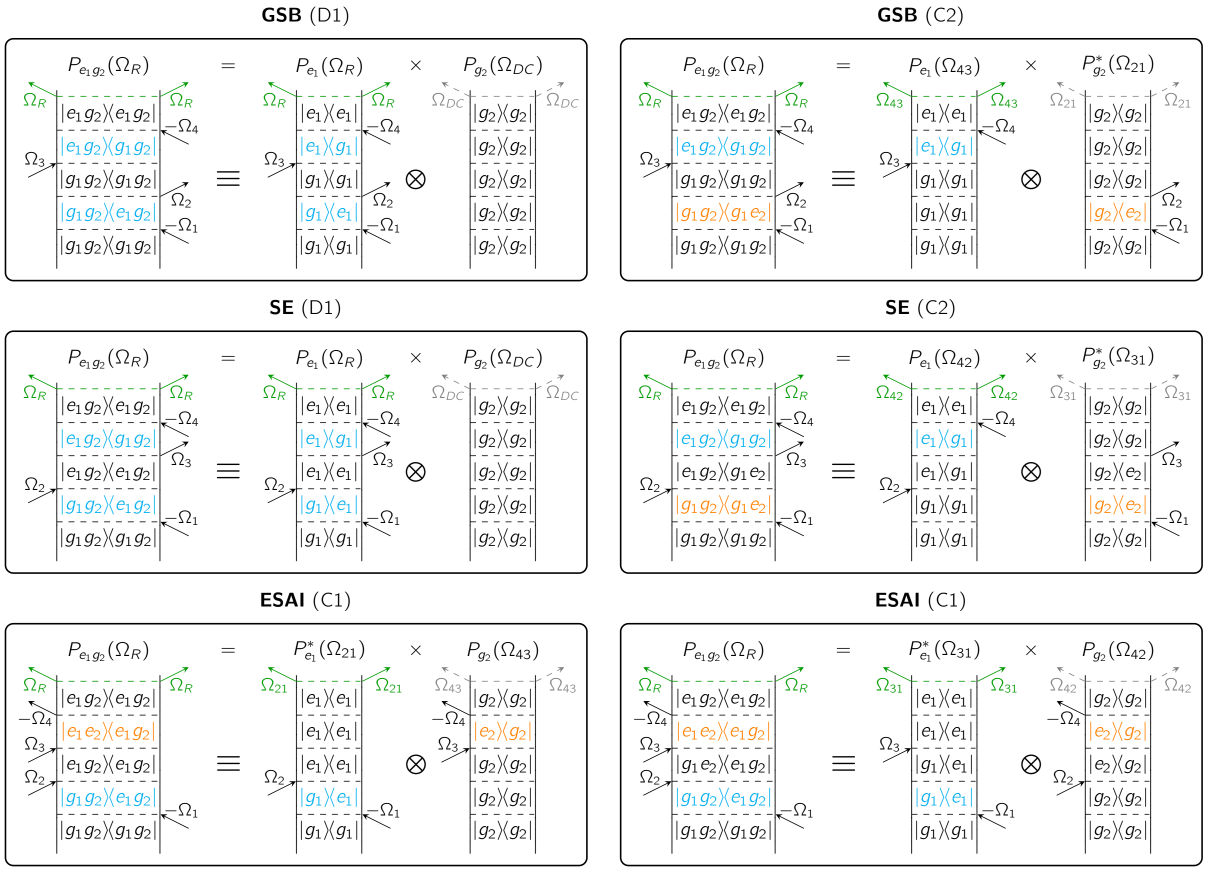

Fig. S1: Feynman diagrams for the rephasing signal emitted from $P_{e_1g_2}$ population in the one- and two-particle representations.

response theory (Sec. 3). As a result of the kinetic scheme, we obtain the time-resolved populations along the detection-time T_d. These can then be further integrated to obtain the time-integrated populations which are used to construct the total signal.

As presented in the main text, the kinetic scheme is defined by the following rates: α_n is the exciton recombination rate, β_n and γ_{12} are the rates of exciton-exciton annihilation leading respectively to the annihilation of one and two excitons. Then, the incoherent signal is defined by the emission rates of the states which are Γ_1, Γ_2 and Γ_{12}.

4.1 Two-Particle Populations

The following kinetic scheme for the two-particle populations is assumed:

$$\begin{cases} \dfrac{d}{dt}P_{e_1g_2}(t) = -\alpha_1 P_{e_1g_2}(t) + (\alpha_2+\beta_2)P_{e_1e_2}(t) \\ \dfrac{d}{dt}P_{g_1e_2}(t) = -\alpha_2 P_{g_1e_2}(t) + (\alpha_1+\beta_1)P_{e_1e_2}(t) \\ \dfrac{d}{dt}P_{e_1e_2}(t) = -(\alpha_1+\beta_1+\alpha_2+\beta_2+\gamma_{12})P_{e_1e_2}(t) \end{cases} \tag{23}$$

whose solution gives the time-resolved populations:

$$\begin{cases} P_{e_1g_2}(t) = P_{e_1g_2}(0)e^{-\alpha_1 t} + \dfrac{\alpha_2+\beta_2}{\beta_1+\alpha_2+\beta_2+\gamma_{12}}P_{e_1e_2}(0)e^{-\alpha_1 t}\left(1-e^{-(\beta_1+\alpha_2+\beta_2+\gamma_{12})t}\right) \\ P_{g_1e_2}(t) = P_{g_1e_2}(0)e^{-\alpha_2 t} + \dfrac{\alpha_1+\beta_1}{\alpha_1+\beta_1+\beta_2+\gamma_{12}}P_{e_1e_2}(0)e^{-\alpha_2 t}\left(1-e^{-(\alpha_1+\beta_1+\beta_2+\gamma_{12})t}\right) \\ P_{e_1e_2}(t) = P_{e_1e_2}(0)e^{-(\alpha_1+\beta_1+\alpha_2+\beta_2+\gamma_{12})t} \end{cases} \tag{24}$$

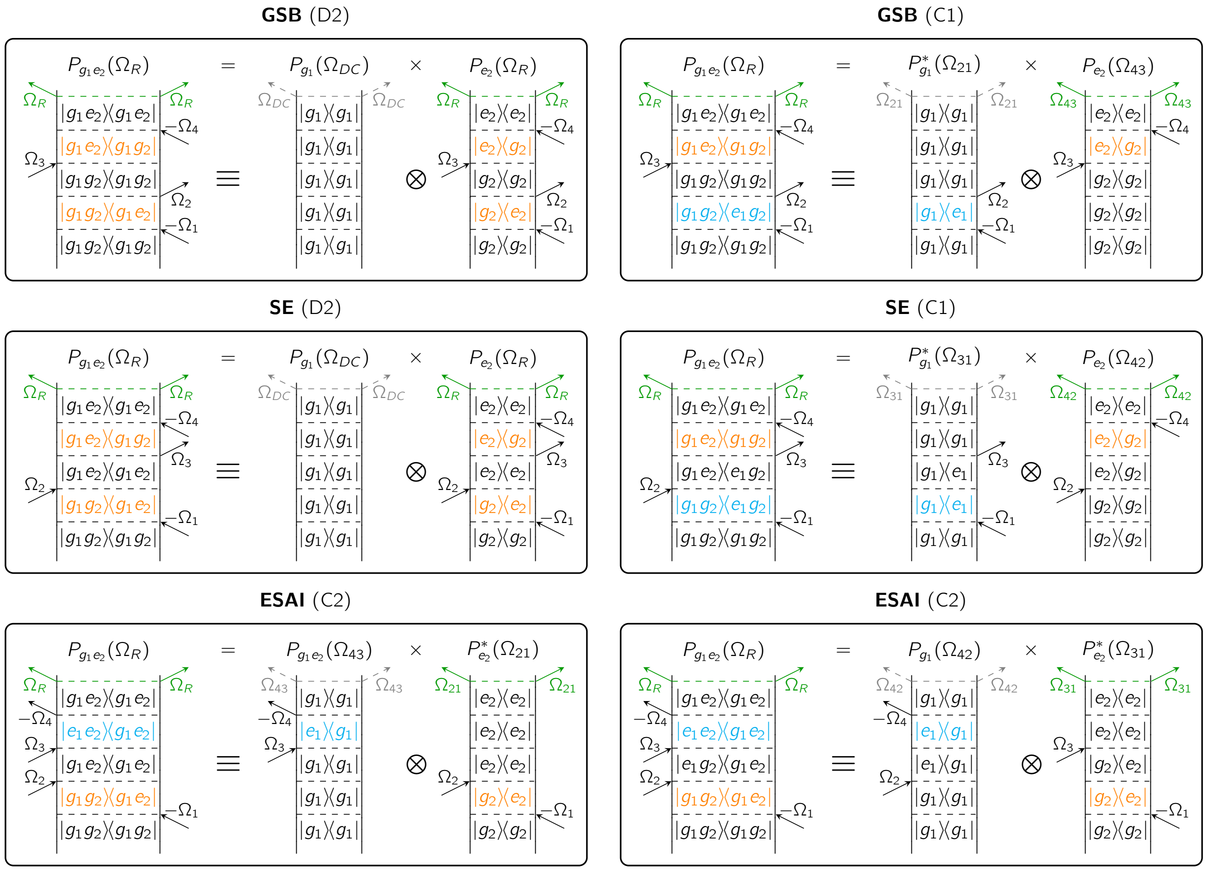

Fig. S2: Feynman diagrams for the rephasing signal emitted from $P_{g_1e_2}$ population in the one- and two-particle representations.

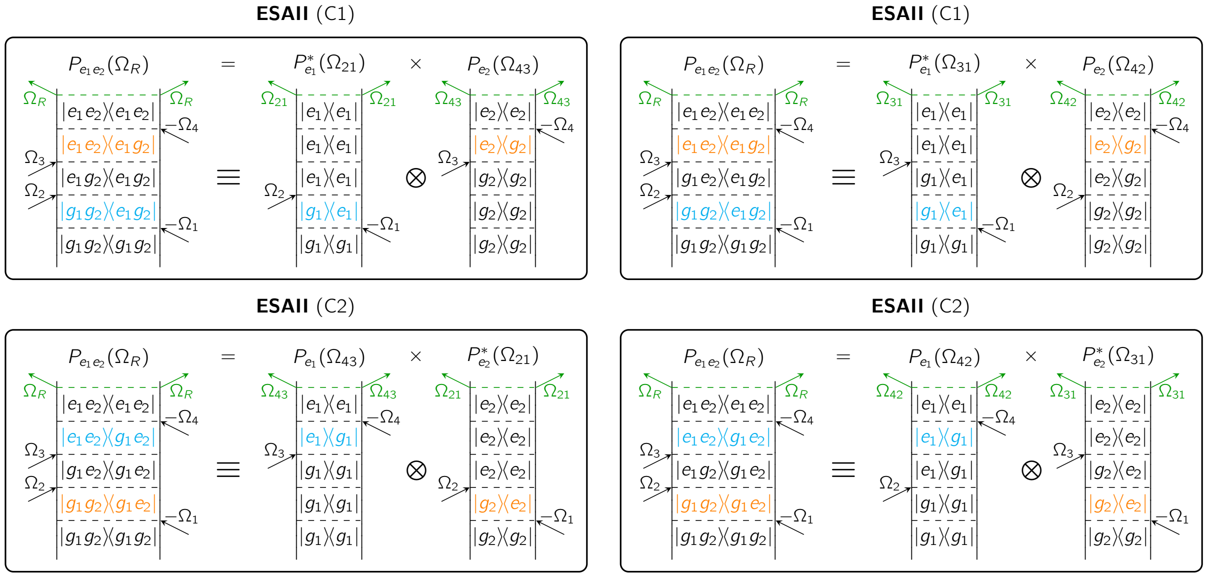

Fig. S3: Feynman diagrams for the rephasing signal emitted from $P_{e_1e_2}$ population in the one- and two-particle representations.

with $P_{e_1g_2}(0)$, $P_{g_1e_2}(0)$ and $P_{e_1e_2}(0)$ as initial conditions. The time-resolved signal along the detection-time T_d is defined on the basis of two-particle populations as:

$$\mathcal{S}(T_d) = \Gamma_1 P_{e_1g_2}(T_d) + \Gamma_2 P_{g_1e_2}(T_d) + \Gamma_{12} P_{e_1e_2}(T_d). \tag{25}$$

By integrating the time-resolved populations for finite detection-time T_d, the time-gated populations are obtained:

$$\overline{P_{e_1g_2}(T_d)} = P_{e_1g_2}(0)\frac{1-e^{-\alpha_1 T_d}}{\alpha_1} - \frac{\alpha_2+\beta_2}{\beta_1+\alpha_2+\beta_2+\gamma_{12}}P_{e_1e_2}(0)\left(\frac{1-e^{-(\alpha_1+\beta_1+\alpha_2+\beta_2+\gamma_{12})T_d}}{\alpha_1+\beta_1+\alpha_2+\beta_2+\gamma_{12}} - \frac{1-e^{-\alpha_1 T_d}}{\alpha_1}\right)$$
$$\overline{P_{g_1e_2}(T_d)} = P_{g_1e_2}(0)\frac{1-e^{-\alpha_2 T_d}}{\alpha_2} - \frac{\alpha_1+\beta_1}{\alpha_1+\beta_1+\beta_2+\gamma_{12}}P_{e_1e_2}(0)\left(\frac{1-e^{-(\alpha_1+\beta_1+\alpha_2+\beta_2+\gamma_{12})T_d}}{\alpha_1+\beta_1+\alpha_2+\beta_2+\gamma_{12}} - \frac{1-e^{-\alpha_2 T_d}}{\alpha_2}\right)$$
$$\overline{P_{e_1e_2}(T_d)} = P_{e_1e_2}(0)\frac{1-e^{-(\alpha_1+\beta_1+\alpha_2+\beta_2+\gamma_{12})T_d}}{\alpha_1+\beta_1+\alpha_2+\beta_2+\gamma_{12}} \tag{26}$$

which can be used to calculate the time-gated signal:

$$\overline{\mathcal{S}(T_d)} = \Gamma_1\overline{P_{e_1g_2}(T_d)} + \Gamma_2\overline{P_{g_1e_2}(T_d)} + \Gamma_{12}\overline{P_{e_1e_2}(T_d)}. \tag{27}$$

By fully integrating along the detection-time T_d, the time-integrated populations are obtained:

$$\begin{cases} \overline{P_{e_1g_2}} = \dfrac{1}{\alpha_1}P_{e_1g_2}(0) + \dfrac{\alpha_2+\beta_2}{\alpha_1(\alpha_1+\beta_1+\alpha_2+\beta_2+\gamma_{12})}P_{e_1e_2}(0) \\ \overline{P_{g_1e_2}} = \dfrac{1}{\alpha_2}P_{g_1e_2}(0) + \dfrac{\alpha_1+\beta_1}{\alpha_2(\alpha_1+\beta_1+\alpha_2+\beta_2+\gamma_{12})}P_{e_1e_2}(0) \\ \overline{P_{e_1e_2}} = \dfrac{1}{\alpha_1+\beta_1+\alpha_2+\beta_2+\gamma_{12}}P_{e_1e_2}(0) \end{cases} \tag{28}$$

which are used to define the time-integrated signal:

$$\begin{aligned} \overline{\mathcal{S}} &= \Gamma_1\overline{P_{e_1g_2}} + \Gamma_2\overline{P_{g_1e_2}} + \Gamma_{12}\overline{P_{e_1e_2}} \\ &= \frac{\Gamma_1}{\alpha_1}P_{e_1g_2}(0) + \frac{\Gamma_2}{\alpha_2}P_{g_1e_2}(0) + \frac{\Gamma_1\alpha_2(\alpha_2+\beta_2)+\Gamma_2\alpha_1(\alpha_1+\beta_1)+\Gamma_{12}\alpha_1\alpha_2}{\alpha_1\alpha_2(\alpha_1+\beta_1+\alpha_2+\beta_2+\gamma_{12})}P_{e_1e_2}(0) \\ &= \Phi_1 P_{e_1g_2}(0) + \Phi_2 P_{g_1e_2}(0) + \big(\Phi_1\cdot\Pi_{e_1e_2\to e_1g_2} + \Phi_2\cdot\Pi_{e_1e_2\to g_1e_2} + \Phi_{12}\big)P_{e_1e_2}(0) \end{aligned} \tag{29}$$

where $\Phi_1 = \frac{\Gamma_1}{\alpha_1}$ and $\Phi_2 = \frac{\Gamma_2}{\alpha_2}$ are the quantum yields of the one-exciton states, $\Phi_{12} = \frac{\Gamma_{12}}{\alpha_1+\beta_1+\alpha_2+\beta_2+\gamma_{12}}$ is the quantum yield of the two-exciton state, while $\Pi_{e_1e_2\to e_1g_2} = \frac{\alpha_2+\beta_2}{\alpha_1+\beta_1+\alpha_2+\beta_2+\gamma_{12}}$ and $\Pi_{e_1e_2\to g_1e_2} = \frac{\alpha_1+\beta_1}{\alpha_1+\beta_1+\alpha_2+\beta_2+\gamma_{12}}$ are the probabilities that the two-exciton state converts to one or the other one-exciton state during the detection-time T_d.

4.2 One-Particle Populations

The equivalent kinetic scheme for the one-particle populations is:

$$\begin{cases} \dfrac{d}{dt}P_{e_1}(t) = -\alpha_1 P_{e_1}(t) - (\beta_1+\gamma_{12})P_{e_1e_2}(t) \\ \dfrac{d}{dt}P_{e_2}(t) = -\alpha_2 P_{e_2}(t) - (\beta_2+\gamma_{12})P_{e_1e_2}(t) \end{cases} \tag{30}$$

which explicitly depends on the two-exciton population $P_{e_1e_2}(t)$. The kinetic scheme can be solved by using the solution for $P_{e_1e_2}(t)$ (Eq. 24) to obtain:

$$\begin{cases} P_{e_1}(t) = P_{e_1}(0)e^{-\alpha_1 t} - \dfrac{\beta_1+\gamma_{12}}{\beta_1+\alpha_2+\beta_2+\gamma_{12}}P_{e_1}(0)\times P_{e_2}(0)e^{-\alpha_1 t}\Big(1-e^{-(\beta_1+\alpha_2+\beta_2+\gamma_{12})t}\Big) \\ P_{e_2}(t) = P_{e_2}(0)e^{-\alpha_2 t} - \dfrac{\beta_2+\gamma_{12}}{\alpha_1+\beta_1+\beta_2+\gamma_{12}}P_{e_1}(0)\times P_{e_2}(0)e^{-\alpha_2 t}\Big(1-e^{-(\alpha_1+\beta_1+\beta_2+\gamma_{12})t}\Big) \end{cases} \tag{31}$$

where we have assumed the initial factorization of the two-exciton population $P_{e_1e_2}(0) = P_{e_1}(0)\times P_{e_2}(0)$ (Eq. 12). By imposing the condition $\Gamma_{12} = \Gamma_1 + \Gamma_2$, the time-resolved signal along the detection-time T_d is defined on the basis of one-particle populations as:

$$\mathcal{S}(T_d) = \Gamma_1 P_{e_1}(T_d) + \Gamma_2 P_{e_2}(T_d). \tag{32}$$

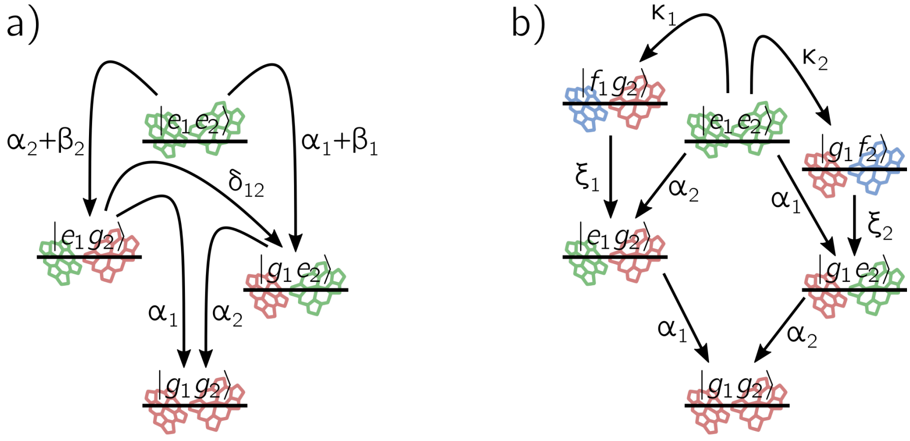

Fig. S4: Alternative kinetic schemes for two-particle populations during the detection-time T_d. Scheme a) includes EET process between one-exciton states at rate δ_{12}. Scheme b) describes EEA process explicitly, accounting for EET between two-exciton state and localized double-excited states at rate κ_n, followed by rapid internal conversion to one-exciton states at rate ξ_n.

By integrating the time-resolved populations for finite detection-time T_d, the time-gated populations are obtained:

$$\overline{P_{e_1}(T_d)} = P_{e_1}(0)\frac{1-e^{-\alpha_1 T_d}}{\alpha_1} - \frac{\beta_1+\gamma_{12}}{\beta_1+\alpha_2+\beta_2+\gamma_{12}} P_{e_1}(0)\times P_{e_2}(0)\left(\frac{1-e^{-\alpha_1 T_d}}{\alpha_1} - \frac{1-e^{-(\alpha_1+\beta_1+\alpha_2+\beta_2+\gamma_{12})T_d}}{\alpha_1+\beta_1+\alpha_2+\beta_2+\gamma_{12}}\right)$$
$$\overline{P_{e_2}(T_d)} = P_{e_2}(0)\frac{1-e^{-\alpha_2 T_d}}{\alpha_2} - \frac{\beta_2+\gamma_{12}}{\alpha_1+\beta_1+\beta_2+\gamma_{12}} P_{e_1}(0)\times P_{e_2}(0)\left(\frac{1-e^{-\alpha_2 T_d}}{\alpha_2} - \frac{1-e^{-(\alpha_1+\beta_1+\alpha_2+\beta_2+\gamma_{12})T_d}}{\alpha_1+\beta_1+\alpha_2+\beta_2+\gamma_{12}}\right) \tag{33}$$

which can be used to calculate the time-gated signal:

$$\overline{S(T_d)} = \Gamma_1 \overline{P_{e_1}(T_d)} + \Gamma_2 \overline{P_{e_2}(T_d)}. \tag{34}$$

By fully integrating along the detection-time T_d, the time-integrated populations are obtained:

$$\begin{cases} \overline{P_{e_1}} = \dfrac{1}{\alpha_1} P_{e_1}(0) - \dfrac{\beta_1+\gamma_{12}}{\alpha_1(\alpha_1+\beta_1+\alpha_2+\beta_2+\gamma_{12})} P_{e_1}(0)\times P_{e_2}(0) \\ \overline{P_{e_2}} = \dfrac{1}{\alpha_2} P_{e_2}(0) - \dfrac{\beta_2+\gamma_{12}}{\alpha_2(\alpha_1+\beta_1+\alpha_2+\beta_2+\gamma_{12})} P_{e_1}(0)\times P_{e_2}(0) \end{cases} \tag{35}$$

which are used to calculate the time-integrated signal:

$$\begin{aligned} \overline{S} &= \Gamma_1 \overline{P_{e_1}} + \Gamma_2 \overline{P_{e_2}} \\ &= \frac{\Gamma_1}{\alpha_1} P_{e_1}(0) + \frac{\Gamma_2}{\alpha_2} P_{e_2}(0) - \frac{\Gamma_1\alpha_2(\beta_1+\gamma_{12}) + \Gamma_2\alpha_1(\beta_2+\gamma_{12})}{\alpha_1\alpha_2(\alpha_1+\beta_1+\alpha_2+\beta_2+\gamma_{12})} P_{e_1}(0)\times P_{e_2}(0) \\ &= \Phi_1 P_{e_1}(0) + \Phi_2 P_{e_2}(0) - \left(\Phi_1\cdot\Pi^{EEA}_{e_1\to g_1} + \Phi_2\cdot\Pi^{EEA}_{e_2\to g_2}\right) P_{e_1}(0)\times P_{e_2}(0) \end{aligned} \tag{36}$$

where $\Pi^{EEA}_{e_1\to g_1} = \frac{\beta_1+\gamma_{12}}{\alpha_1+\beta_1+\alpha_2+\beta_2+\gamma_{12}}$ and $\Pi^{EEA}_{e_2\to g_2} = \frac{\beta_2+\gamma_{12}}{\alpha_1+\beta_1+\alpha_2+\beta_2+\gamma_{12}}$ represent the probabilities to convert from the excited to the ground-state through EEA process during the detection-time T_d.

5 Generalized Kinetic Scheme

In this section, we report a generalized kinetic scheme based on the classical master equation. We consider the system composed by $N+1$ states $|n\rangle$, with $n = 0, 1, \ldots, N$. The state $|n=0\rangle$ represents the collective ground-state, while the remaining N states $|n \neq 0\rangle$ are excited-states.

The populations are assumed to follow the classical master equation:

$$\frac{d}{dt}\vec{P}(t) = -\boldsymbol{K}\vec{P}(t) \tag{37}$$

where $\vec{P}(t) = \sum_{n=0}^{N} P_n(t)\,|n\rangle$ is the population vector, with $P_n(t)$ population of the n-th state, and $\boldsymbol{K}$ is the kinetic matrix whose elements are defined as:

$$K_{nm} = -(1-\delta_{nm})k_{n\leftarrow m} + \delta_{nm}\sum_{l\neq m} k_{l\leftarrow m} \tag{38}$$

where the first terms is responsible for the gain in population from other states $\{|m\rangle\}$ to state $|n\rangle$ with rate $k_{n\leftarrow m}$, while the second term accounts for the loss in population from state $|n\rangle$ to other states $\{|m\rangle\}$ with rate $k_{l\leftarrow m}$. The formal solution of the classical master equation is:

$$\vec{P}(t) = e^{-\boldsymbol{K}t}\vec{P}(0) \tag{39}$$

with population vector $\vec{P}(0)$ as initial condition. By using the spectral decomposition, the kinetic matrix can be written as $\boldsymbol{K} = \boldsymbol{V}\boldsymbol{\Lambda}\boldsymbol{V}^{-1}$, where $\boldsymbol{\Lambda} = \sum_{k=0}^{N} \lambda_k\,|k\rangle\langle k|$ is the eigenvalue matrix and $\boldsymbol{V} = \sum_{n,k} V_{nk}\,|n\rangle\langle k|$ is the eigenvector matrix. Each eigenvector $|k\rangle = \sum_{n=0}^{N} V_{nk}\,|n\rangle$ is associated with a real eigenvalue $\lambda_k \geq 0$. The solution of the master equation can thus be rewritten as:

$$\vec{P}(t) = \boldsymbol{V}e^{-\boldsymbol{\Lambda}t}\boldsymbol{V}^{-1}\vec{P}(0) \tag{40}$$

where the matrix exponential is $e^{-\boldsymbol{\Lambda}t} = \sum_{k=0}^{N} e^{-\lambda_k t}\,|k\rangle\langle k|$.

We now assume that the kinetic scheme admits one equilibrium state corresponding to the eigenstate $|k=0\rangle$ with eigenvalue $\lambda_0 = 0$. In the context of optical spectroscopy, $|k=0\rangle$ coincides with the collective ground-state $|n=0\rangle$. Therefore, the elements of the matrix exponential are:

$$\left[e^{-\boldsymbol{\Lambda}t}\right]_{kk} = \begin{cases} 1 & \text{for } \lambda_0 = 0 \\ e^{-\lambda_k t} & \text{for } \lambda_k > 0 \end{cases} \tag{41}$$

depending on the eigenvalue λ_k. We now introduce the emission matrix $\boldsymbol{\Gamma} = \sum_n \Gamma_n\,|n\rangle\langle n|$, where Γ_n is the emission rate of the n-th state. In the following, we assume that the collective ground-state do not emit signal $\Gamma_0 = 0$, while for the other excited-states $\Gamma_n \geq 0$ in general.

The time-resolved signal vector along the detection-time T_d is defined as:

$$\vec{S}(T_d) = \boldsymbol{\Gamma}\cdot\vec{P}(T_d) \tag{42}$$

with population vector defined in Eq. 40. The time-integrated population vector is given by:

$$\overline{\vec{P}} = \int_0^{\infty} dT_d\;\vec{P}(T_d) = \boldsymbol{V}\left[\int_0^{\infty} dT_d\;e^{-\boldsymbol{\Lambda}T_d}\right]\boldsymbol{V}^{-1}\vec{P}(0) \tag{43}$$

where the integral of the elements of the matrix exponential is:

$$\int_0^{\infty} dT_d\;\left[e^{-\boldsymbol{\Lambda}T_d}\right]_{kk} = \int_0^{\infty} dT_d\;e^{-\lambda_k T_d} = \begin{cases} +\infty & \text{if } \lambda_0 = 0 \\ \frac{1}{\lambda_n} & \text{if } \lambda_k > 0. \end{cases} \tag{44}$$

Therefore, the time-integrated signal vector can be obtained as:

$$\overline{\vec{S}} = \boldsymbol{\Gamma}\cdot\overline{\vec{P}} \tag{45}$$

by inserting the time-integrated population vector (Eq. 43).

6 Chromophoric Pair of Two-Level Systems

In this section, we report additional results regarding the chromophoric pair model of two-level systems.

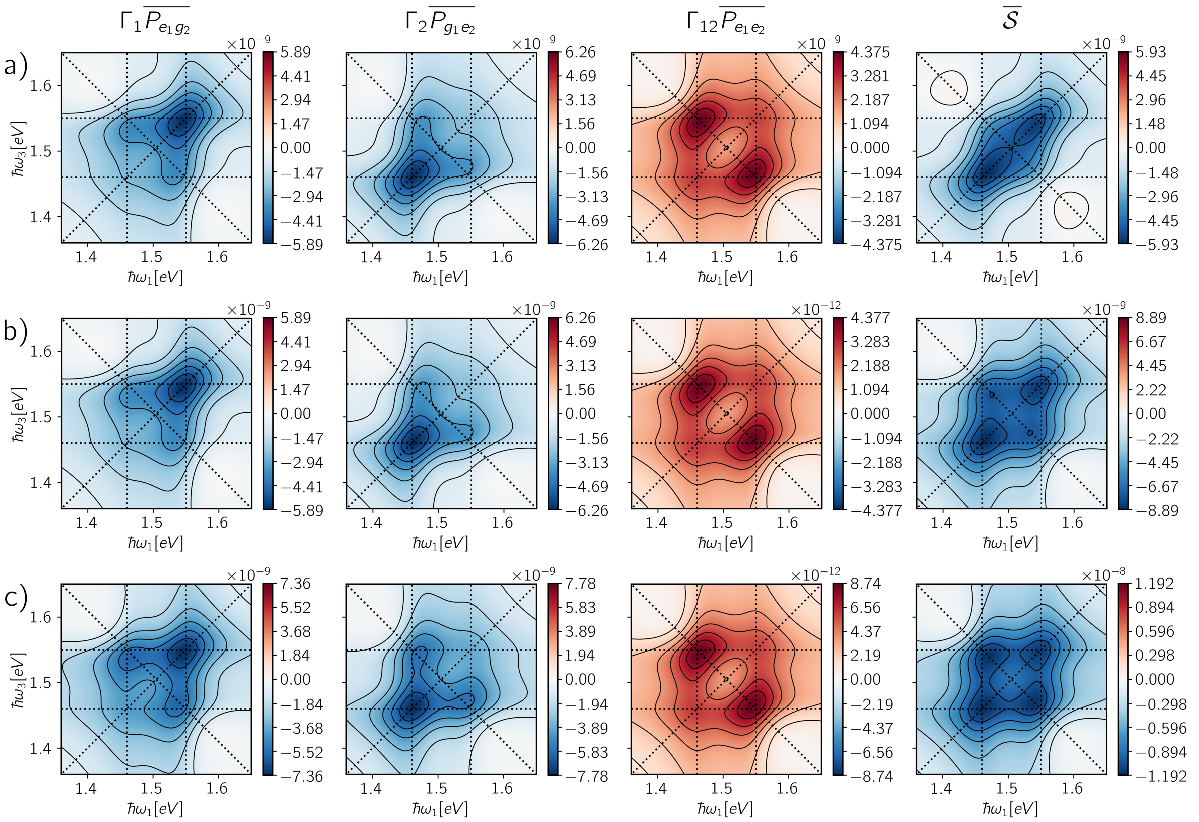

Fig. S5: Time-integrated rephasing spectra corresponding to the contributions appearing in Eq. 29 for different EEA rates: a) $\beta_n^{-1} = 1$ μs and $\gamma_{12}^{-1} = 1$ μs, b) $\beta_n^{-1} = 1$ ps and $\gamma_{12}^{-1} = 1$ μs, c) $\beta_n^{-1} = 1$ μs and $\gamma_{12}^{-1} = 1$ ps. Exciton recombination rates $\alpha_n = 1$ ns^{-1}, emission rates $\Gamma_n = 1$ ns^{-1} and $\Gamma_{12} = \Gamma_1 + \Gamma_2$ are kept constant.

In Fig. S5 are reported the time-integrated spectra corresponding to the total signal and the individual contributions in Eq. 29 for different EEA rates. Notice that, due to the faster EEA rate, the contributions from the biexciton state $\Gamma_{12}\overline{P_{e_1e_2}}$ in Fig. S5b-c are considerably smaller compared to the one in Fig. S5a. Consequently, this causes the incomplete cancellation of pathways leading to the appearance of cross-peaks in the total spectrum $\overline{\mathcal{S}}$ in Fig. S5b-c. In Fig. S6 are reported the Incoherent Mixing Factor, defined by the coefficient of the population product in Eq. 36 and the cross peak amplitude as a function of β_n/α_n for different values of γ_{12}.

Furthermore, we investigated the effects on the spectra of Exciton Energy Transfer (EET) during the detection-time T_d. By using the method outlined in Sec. 5, we introduce a kinetic scheme which includes the EET process $|e_1g_2\rangle \rightarrow |g_1e_2\rangle$ with rate δ_{12}, in addition to EEA, as depicted in Fig. S4a. In Fig. S7 are reported the time-integrated spectra of the individual contributions and the total signal for increasing EET rates δ_{12}. We observe that, while the presence of the EET process does not affect the total spectra, it may affect the individual contributions. Specifically, we notice that pathways ending in $P_{e_1g_2}$ population at $T_d = 0$ fs are transferred to the $P_{g_1e_2}$ population during the detection-time, resulting in their appearance in the corresponding spectra.

In addition, in Fig. S8 are reported the time-integrated spectra for different quantum yields Φ_n of the two chromophores. In addition to the enhancement/depletion of spectral features associated with either chromophore, we point out how variations in the relative quantum yields can also affect the amplitude of cross-peaks.

The considerations drawn in this work for the rephasing signal also apply to other components of the response, i.e., non-rephasing signal ($\Omega_{NR} = +\Omega_1 - \Omega_2 + \Omega_3 - \Omega_4$) and double-quantum coherence signal ($\Omega_{DQC} = +\Omega_1 + \Omega_2 - \Omega_3 - \Omega_4$). As a result of the non-perturbative protocol, these contributions are readily available from our simulations. For sake of completeness, the non-rephasing spectra from the one-

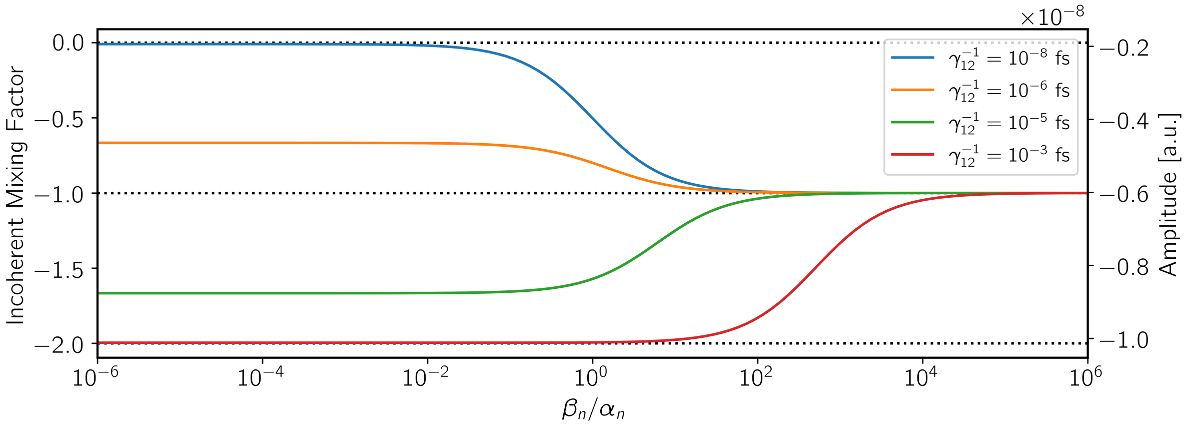

Fig. S6: Dependence of the Incoherent Mixing Factor in Eq. 36 and cross-peaks amplitude for different values of EEA rates β_n, and γ_{12}. Exciton recombination rates $\alpha_n = 1$ ns^{-1}, emission rates $\Gamma_n = 1$ ns^{-1} and $\Gamma_{12} = \Gamma_1 + \Gamma_2$ are kept constant.

and two-particle populations at the end of the pulse train are reported Fig. S9. Due to the complete lack of time-ordering between the second and the third pulses at $T_2 = 0$ fs, double-quantum coherence signal results identical to the non-rephasing signal.

7 Chromophoric Pair of Three-Level Systems

In this section, we extend the chromophoric pair model by treating each chromophore as a three-level system, i.e., composed by a ground state $|g_n\rangle$, a one-exciton state $|e_n\rangle$ and a two-exciton state $|f_n\rangle$. The energy of two-exciton states is assumed to be twice the energy of one-exciton states, with $\epsilon_{f_1} = 3.10$ eV and $\epsilon_{f_2} = 2.92$ eV, respectively. The possible two-particle states are as reported in Fig. S4

In Fig. S10 are reported the additional Feynman diagrams for the rephasing signal, corresponding to ESAI and ESAII self-population pathways. In Fig. S11 are reported the spectra from two-particle populations at $T_d = 0$ fs for states in the one-exciton manifold (Fig. S11a-b), two-exciton manifold (Fig. S11c-e), three-exciton manifold (Fig. S11g-h) and four-exciton manifold (Fig. S11i). Notice how the spectra from the states in the three- and four-exciton manifolds have smaller amplitudes compared to the others, as they arise from higher-order contributions in the light-matter interaction. For simplicity, we neglect those contributions in the definition of the time-integrated signal:

$$\overline{\mathcal{S}} = \Gamma_{e_1}\overline{P_{e_1 g_2}} + \Gamma_{e_2}\overline{P_{g_1 e_2}} + (\Gamma_{e_1} + \Gamma_{e_2})\overline{P_{e_1 e_1}} + \Gamma_{f_1}\overline{P_{f_1 g_2}} + \Gamma_{f_2}\overline{P_{g_1 f_2}}. \tag{46}$$

where the time-integrated populations are weighted by the emission rate of the states.

By using the generalized kinetic scheme reported in Sec. 5, the time-integrated spectra for different emission rates of the two-exciton states Γ_{f_n} compared to the one-exciton states Γ_{e_n} are reported in Fig. S12. We notice that, as $\Gamma_{f_n} > \Gamma_{e_n}$, positive spectral features start to arise in the spectra: diagonal-peaks result from ESAII self-population pathways (Fig. S10), appearing in Fig. S11d-e, while cross-peaks are related to ESAII cross-population pathways (Fig. S3), appearing in Fig. S11c, which can be regarded as incoherent mixing contributions. We point out that fast radiative processes from higher excited-states are not usually present in molecular systems but they may be realized in nanostructures especially in photocurrent-detection.

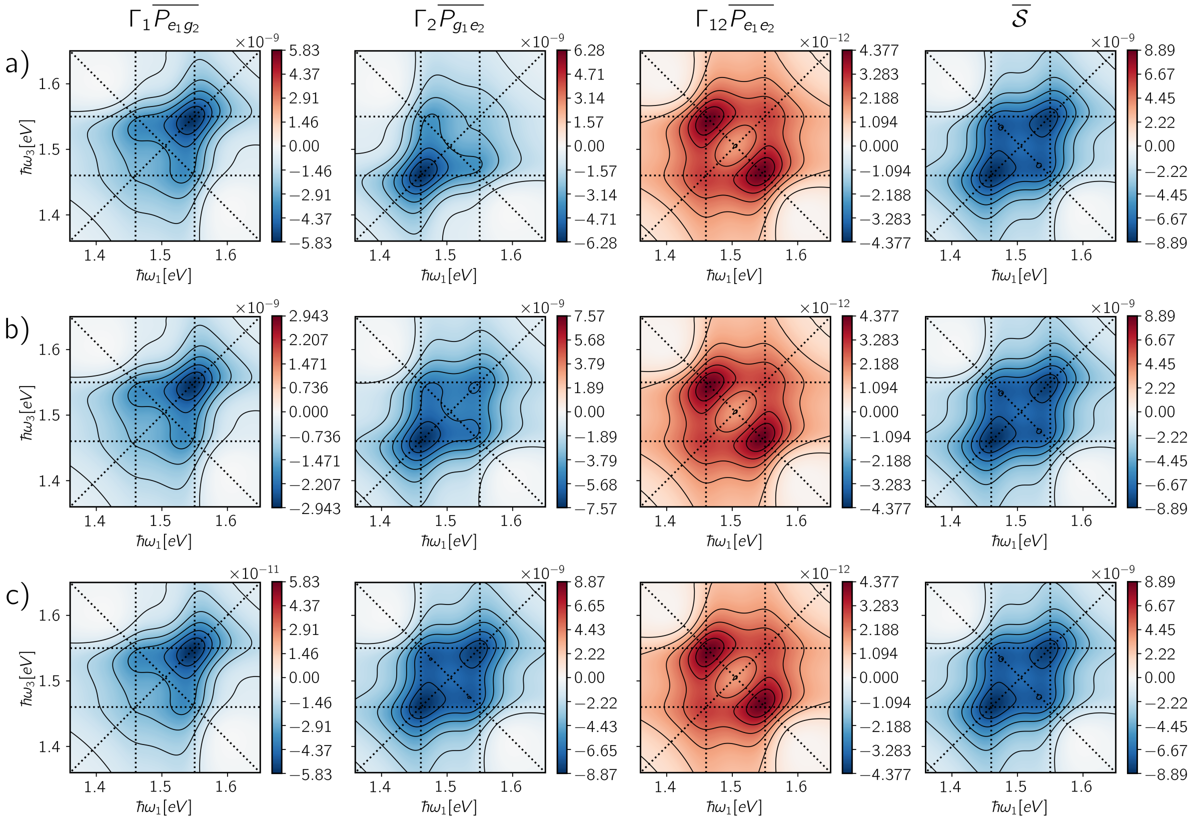

Fig. S7: Time-integrated rephasing spectra obtained using Eq. 43 for different EET rates: a) $\delta_{12}^{-1} = 100$ ns, b) $\delta_{12}^{-1} = 1$ ns, c) $\delta_{12}^{-1} = 10$ ps. Exciton recombination rates $\alpha_n^{-1} = 10$ ns, EEA annihilation rates $\beta_n^{-1} = 1$ ps and $\gamma_{12}^{-1} = 1$ μs, and emission rates $\Gamma_n = 1$ ns^{-1} and $\Gamma_{12} = \Gamma_1 + \Gamma_2$ are kept constant.

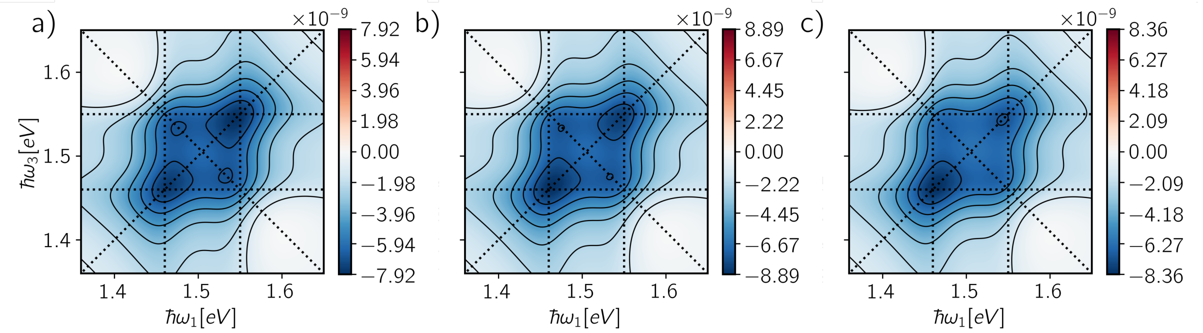

Fig. S8: Time-integrated rephasing spectra for different emission rates: a) $\Gamma_1^{-1} = 0.8$ ns and $\Gamma_2^{-1} = 1.0$ ns, b) $\Gamma_1^{-1} = 1.0$ ns and $\Gamma_2^{-1} = 1.0$ ns, c) $\Gamma_1^{-1} = 1.0$ ns and $\Gamma_2^{-1} = 0.8$ ns, assuming $\Gamma_{12} = \Gamma_1 + \Gamma_2$. Exciton recombination rates $\alpha_n^{-1} = 1$ ns, EEA rates $\beta_n^{-1} = 1$ ps and $\gamma_{12}^{-1} = 1$ μs are kept constant.

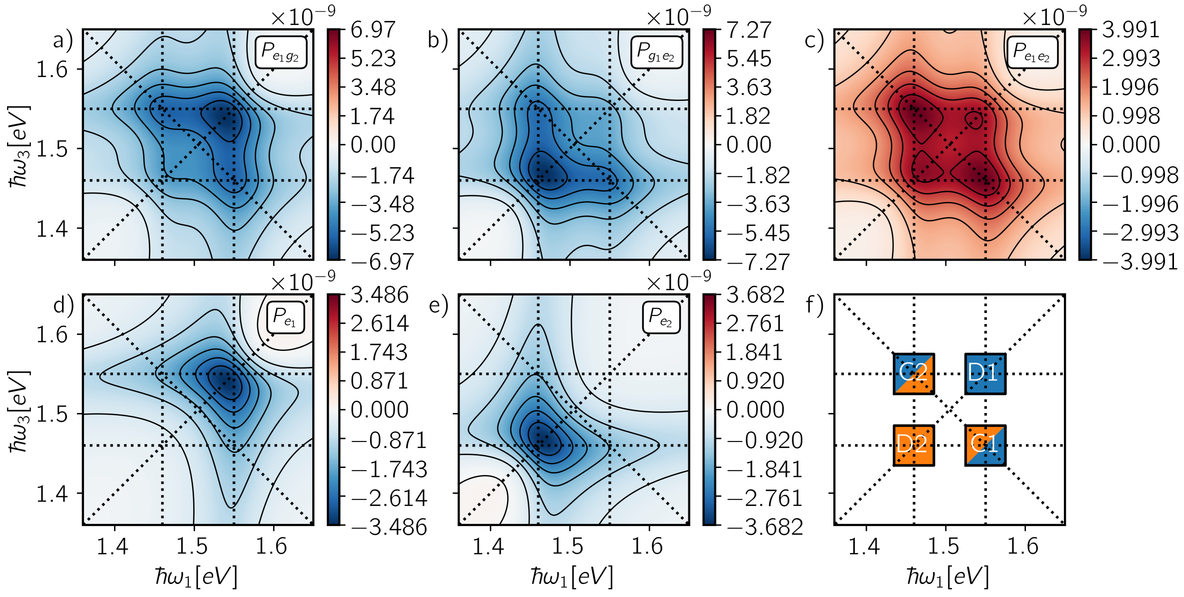

Fig. S9: Non-Rephasing at $T_2 = 0$ fs. This is identical to the Double-Quantum Coherence signal because at $T_2 = 0$ fs there is complete lack of time-ordering.

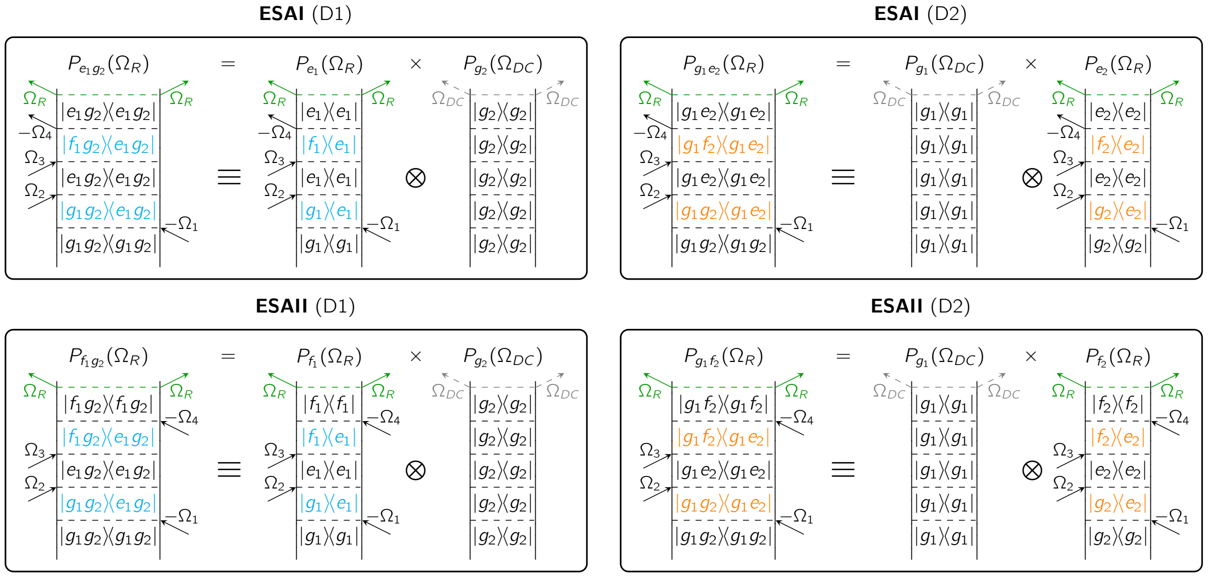

Fig. S10: (left) Feynman diagrams for the rephasing signal modulated at Ω_R emitted from the two-particle population $P_{e_1e_2}$ and (right) the equivalent Feynman diagrams for the one-particle populations.

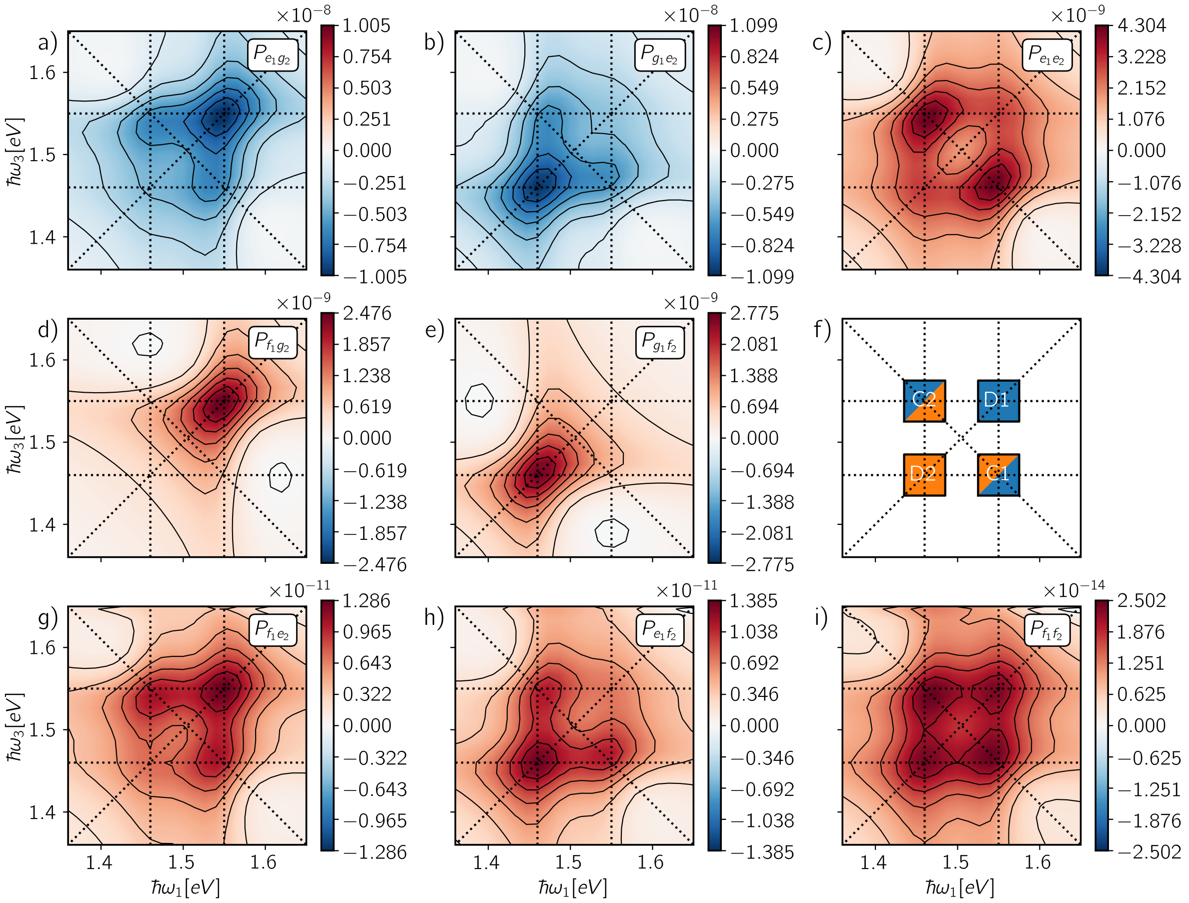

Fig. S11: Rephasing spectra from two-particle populations in the one-exciton manifold a) $P_{e_1 g_2}$, b) $P_{g_1 e_2}$, and two-exciton manifold c) $P_{e_1 e_2}$, d) $P_{f_1 g_2}$, e) $P_{g_1 f_2}$, at detection-time $T_d = 0$ fs. In f) are reported the spectral positions of diagonal peaks (D1 and D2) and cross peaks (C1 and C2). We also report the two-particle populations for the three-exciton manifold g) $P_{f_1 e_2}$, h) $P_{e_1 f_2}$ and for the four-exciton manifold i) $P_{f_1 f_2}$.

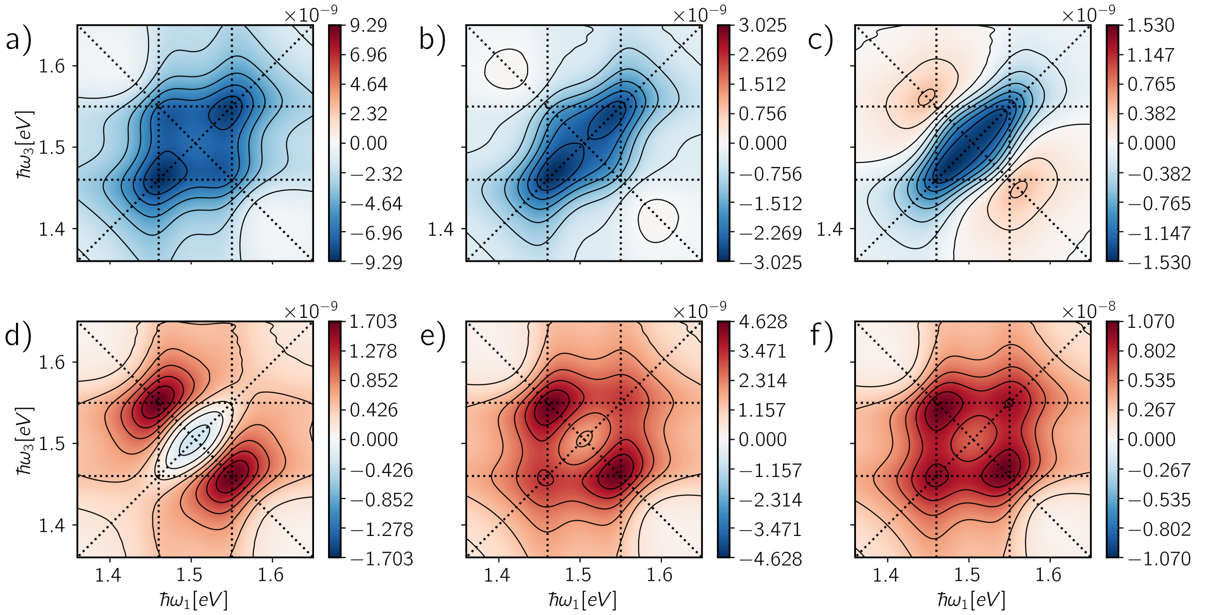

Fig. S12: Time-integrated rephasing spectra for different $\Gamma_{f_n}/\Gamma_{e_n}$ ratios: a) 0.0, b) 1.0, c) 1.25, d) 1.5, e) 2.0, f) 3.0. The emission rate of the single-excited state $\Gamma_{e_n} = 1$ ns^{-1} is kept fixed.

References

(1) Damtie, F. A.; Wacker, A.; Pullerits, T.; Karki, K. J. *Phys. Rev. A* **2017**, *96*, 053830.

(2) Bruschi, M.; Gallina, F.; Fresch, B. *Phys. Chem. Chem. Phys.* **2022**, *24*, 27645–27659.

(3) Kiessling, A. J.; Cina, J. A. *J. Chem. Phys.* **2020**, *152*, 244311.
