# Supplementary material for: Unifying Nonlinear Response and Incoherent Mixing in Action-2D Electronic Spectroscopy
Source: J Phys Chem Lett. 2023 Jul 25;14(30):6872–9. doi: 10.1021/acs.jpclett.3c01670 (PMC10405272; doi:10.1021/acs.jpclett.3c01670)
Supplement: Supplementary file 2 — jz3c01670_si_002.pdf [file jz3c01670_si_002.pdf]

jz-2023-01670u.R1

Name: Peer Review Information for "Unifying Non-Linear Response and Incoherent Mixing in Action-2D Electronic Spectroscopy"

First Round of Reviewer Comments

Reviewer: 1
Comments to the Author
Action-detected two-dimensional electronic spectroscopy (A-2DES) is a rapidly developing class of nonlinear spectroscopy techniques that has already shown tremendous wealth of applications. In order to appreciate the importance of the paper by Bruschi et al., a brief background on the state of A-2DES is needed.
The signal in A-2DES is proportional to the nonlinear excited population bearing the appropriate phase signature resulting from interaction with the excitation pulses. The nonlinear population can arise from interaction with all four pulses, in which case it probes the direct nonlinear response. Or, in contrast to coherent 2DES, two linear populations can mix during the signal emission by a nonlinear process such as exciton–exciton annihilation. This has been called 'incoherent mixing' in literature. The scientists in the field of A-2DES can be divided into two camps. The first one typically works with relatively small systems and formulates the response of the whole system in a collective-state basis including multiple excitations. In this picture, the equations of the system are linear and there is no 'incoherent mixing'. The second camp, typically working with extended systems, describes their sample in the single-excitation basis, with nonlinear equations for processes such as excitation interaction. These then lead to the 'incoherent' mixing.
After this somewhat lengthy introduction, we are in the position to appreciate the paper by Bruschi et al.. In their work, the authors bring the two camps together, unifying their language and demonstrating that the difference in their descriptions is, to a large degree, just a matter of language and convenience. These are the formative years, in which the field of action-detected coherent spectroscopy is being established. Sadly, the two camps found little common language so far, and the crack between them threatens to widen even more. This is why the paper "Unifying Non-Linear Response and Incoherent Mixing in Action-2D Electronic Spectroscopy" is timely, very important and definitely should be published.
That said, there are couple of points I feel the authors should take into account before publishing.
1) The identification of 'nonlinear response vs incoherent mixing' with 'multi-particle vs single-particle picture' is not general and neither is the distinction between 'incoherent mixing' and 'nonlinear response' based on nonlinear processes.
I completely agree with the authors that what is described in single-particle picture as incoherent mixing can be described in two-particle picture as nonlinear response. However, the paper (see, e.g., abstract), reads such that the need for two-particle description of non-linear response is identified with presence of incoherent mixing. In case of four-wave mixing (fourth-order perturbation theory in density matrix), this is indeed so, since the nonlinear processes such as exciton–exciton annihilation (EEA) take place only after the interaction with the four

pulses, because only then can two excitations be present in the system. Consider, however, higher-order response such as six-wave mixing (see, e.g., work by the Brixner group). There, EEA can happen after the four interactions with the first two pulses, being part of the ‘true’ nonlinear response of the sample.
2) Drawing the line based on ‘system of interest’ (p. 18) seems somewhat arbitrary.
How does one draw a line between systems that can still ‘mix’ within their ‘true’ nonlinear response, and those who mix ‘incoherently’? This should be based at least on some separation of timescales, compared to the time it takes to produce the incoherent signal. However, even this seems difficult – would then what is termed ‘incoherent mixing’ in fluorescence-detected 2DES be ‘true nonlinear response’ under photocurrent detection?
3) The terminology of ‘incoherent mixing’ is deceptive.
I realize that the authors did not invent this term, but their manuscript presents an opportunity to clarify it. In its original use, the term was probably supposed to contrast with the ‘coherent’ nonlinear response. However, as the authors point out, this is to a large degree just a matter of representation. Incoherent mixing would, by definition, imply mixing without preserving the phase. However, the mixed populations do preserve the combined phase, that is why they are extracted together with the nonlinear response signal.
4) Possible better distinction between mixing and nonlinear response?
The authors provide ideas how to distinguish the ‘incoherent mixing’ and ‘nonlinear response’. In light of points 1) and 2) and what authors write on page 18 about the choice of representation, would it be possible to establish a general, system-independent definition of ‘nonlinear response’ and ‘signal mixing’. Would, as a suggestion, simply defining ‘whatever happens between the pulses’ as ‘nonlinear response’, and ‘whatever happens during signal emission’ as ‘signal mixing’?
5) The ‘incoherent mixing’ can be distinguished and probed by gating the signal.
Several works, including that of the authors (references 25, 35 of the manuscript) have studied dependence of the mixing (typically in the form of spectral cross peaks) on the time of the signal emission. Indeed, if one would gate the signal immediately after the interaction with the last pulse, there should be no time for nonlinear population mixing, and the signal should consist of the nonlinear response only. (This motivates the proposed definition in point 5). The authors could comment on the possibility of signal gating in manipulating the incoherent mixing part of the signal.

Reviewer: 2
Comments to the Author
The manuscript deals with interpretational issues of the action detected 2D electronic spectroscopy. The action-detected coherent spectroscopy combines the advantages of fully coherent methods with the possibly higher detection sensitivity of the action detection schemes such as fluorescence detection. Fluorescence-detected 2D electronic spectroscopy ideally reports on the same processes and electronic structure relations as the coherently detected version of the method, however, it has been shown that exciton-exciton annihilation deletes certain signal components. Most notably, there are cross-peaks at waiting time T=0 in the fluorescence-detected spectra of a weakly coupled dimer, while no cross-peaks appear in the coherently detected spectra.

The cross-peaks in the action detected 2D spectra caused confusion in some early publications and there were various claims about them reporting interactions between molecules in the same way as the coherently detected methods. One reason for this confusion was apparent alternative explanations of the origin of the signals occurring in the method. The present paper attempts to reconcile the alternatives and show that in the weak coupling limit, they provide equivalent descriptions.

I have to admit that I come completely from the non-linear response side of the story. Non-linear response theory explains all the signals in both detection versions of the spectroscopy and does that both in the weak and strong interaction limits. This is the reason why I always hesitated to spend extensive time learning the background of the incoherent mixing description. The value of the present paper for me is in bringing closer to attention some of the advantages of the alternative. The authors make a good job of comparing the two descriptions, pointing out their similarities and differences. It is important that they link the two descriptions with the one-particle and two-particle descriptions. From this linking it is immediately clear that there might be practical advantages in treating weakly coupled systems with the incoherent mixing picture.

I have no doubt that this paper is a valuable addition to the literature, clarifying an important interpretational issue and also pointing towards its practical combination with non-linear response theory. I think the paper can be published with the minor adjustments that I suggest below.

I have two suggestions:

1) The paper is overall well-written and structured. At a few points, the wording isn't very clear and I suggest that the authors check their English again to make the text more clear.

2) I suggest that the authors use their insight into both alternative explanations of the signals to clarify a seemingly unimportant etymological issue with the term incoherent mixing. I explain briefly, why I think this is important: The initial confusion about the nature of the cross-peaks might have been related to the fact that the word "mixing" has at least two meanings within the spectroscopy of excitons. Cross-peaks in the coherent 2D spectra report on "excitonic-mixing", i.e. the fact that the observed states are superpositions of local states. This is often interpreted as the presence of coherence and hence it is the "coherent mixing". Clearly the term "incoherent mixing" suggests very much some different type of mixing of exciton states, and I believe that in some early publications (or attempted publications) this idea was used. Nevertheless, there is a different meaning of "mixing" also at play, and this is the mixing of signals, such as in "four-wave mixing". The mixing of signals has nothing to do with the properties of the studied molecular system. Now, which of the two meanings, if any, is closer to the nature of the incoherent mixing discussed in this paper? Clearly, incoherent mixing is not a mixing of excitonic states as it stems from the processes occurring during the detection time. It

would be very useful to the reader (and this referee) if the authors could make a comment in the paper on the nature of incoherent mixing along these lines.

Author's Response to Peer Review Comments:

Padova, 13/07/2023

Dear Prof. Editor

Editor of The Journal of Physical Chemistry Letters

We thank you for your letter and the two reviewers for their positive assessment of our work.

The reviewers' insightful suggestions led us to introduce limited but important changes to our manuscript which I now submit to your attention in its revised version. Attached to this letter, you will find our response to the reviewers' comment also reporting the main changes introduced in the manuscript.

Concerning the non-scientific changes listed in your letter, we ask, if possible, to avoid the explicit spelling of 2D (two-dimensional) in the title of the manuscript: "Unifying Non-Linear Response and Incoherent Mixing in Action-2D Electronic Spectroscopy". Because of its widespread use, we believe the term "2D Electronic Spectroscopy" is the most suitable to unambiguously identify the spectroscopic technique which is the subject of our analysis. All the other changes have been implemented as required by the editorial office.

We hope that you will find the revised version of this manuscript suitable for publication in The Journal of Physical Chemistry Letters.

Yours sincerely,

Prof. Barbara Fresch

On behalf of all the authors:

Matteo Bruschi, Luca Bolzonello, Federico Gallina, Barbara Fresch.

Authors' reply

First of all, we truly thank both reviewers for their supportive comments and stimulating suggestions. We mostly agree with the point of view expressed in their reports, substantially calling for an effort to better define the borders and the physical content of the phenomenon known as incoherent mixing in action-detected spectroscopy.

Following their insightful remarks, we have further elaborated on few important points like 1) the relation between "one and two-particle representations" and the characteristic of the physical system under study and 2) the conditions under which the response can be "tagged" as incoherent mixing. To do so, we have changed/integrated mainly the last part of the manuscript, where discussion and conclusion are drawn.

In the following, we briefly address point by point the reviewer' comments.

Reviewer: 1

Recommendation: This paper is publishable subject to minor revisions noted. Further review is not needed.

Comments:

Action-detected two-dimensional electronic spectroscopy (A-2DES) is a rapidly developing class of nonlinear spectroscopy techniques that has already shown tremendous wealth of applications. In order to appreciate the importance of the paper by Bruschi et al., a brief background on the state of A-2DES is needed.

The signal in A-2DES is proportional to the nonlinear excited population bearing the appropriate phase signature resulting from interaction with the excitation pulses. The nonlinear population can arise from interaction with all four pulses, in which case it probes the direct nonlinear response. Or, in contrast to coherent 2DES, two linear populations can mix during the signal emission by a nonlinear process such as exciton–exciton annihilation. This has been called 'incoherent mixing' in literature. The scientists in the field of A-2DES can be divided into two camps. The first one typically works with relatively small systems and formulates the response of the whole system in a collective-state basis including multiple excitations. In this picture, the equations of the system are linear and there is no 'incoherent mixing'. The second camp, typically working with extended systems, describes their sample in the single-excitation basis, with nonlinear equations for processes such as excitation interaction. These then lead to the 'incoherent' mixing.

After this somewhat lengthy introduction, we are in the position to appreciate the paper by Bruschi et al. In their work, the authors bring the two camps together, unifying their language and demonstrating that the difference in their descriptions is, to a large degree, just a matter of language and convenience. These are

the formative years, in which the field of action-detected coherent spectroscopy is being established. Sadly, the two camps found little common language so far, and the crack between them threatens to widen even more. This is why the paper "Unifying Non-Linear Response and Incoherent Mixing in Action-2D Electronic Spectroscopy" is timely, very important and definitely should be published.

The referee frames the context of our work very effectively. We have integrated in the main text an explicit comment on the relation between the nature of the investigated systems, i.e. extended solid state samples or small molecular aggregates, and the typical choice of the basis to represent the action response. At page 16-17:

"Whereas in the one-particle representation, the focus is on the single chromophore and cross peaks arise from the spurious interaction with another system, the two-particle representation supports the dimeric nature of the system, even when the interaction is weak, and cross peaks are part of the non-linear response of the system as a whole. In the literature about A-2DES, the former view has been traditionally adopted to interpret the response of extended solid-state systems,[31,42] while the latter has been privileged for analyzing the response of small molecular aggregates.[26–28]"

That said, there are couple of points I feel the authors should take into account before publishing.

1) The identification of 'nonlinear response vs incoherent mixing' with 'multi-particle vs single-particle picture' is not general and neither is the distinction between 'incoherent mixing' and 'nonlinear response' based on nonlinear processes.

I completely agree with the authors that what is described in single-particle picture as incoherent mixing can be described in two-particle picture as nonlinear response. However, the paper (see, e.g., abstract), reads such that the need for two-particle description of non-linear response is identified with presence of incoherent mixing. In case of four-wave mixing (fourth-order perturbation theory in density matrix), this is indeed so, since the nonlinear processes such as exciton–exciton annihilation (EEA) take place only after the interaction with the four pulses, because only then can two excitations be present in the system. Consider, however, higher-order response such as six-wave mixing (see, e.g., work by the Brixner group). There, EEA can happen after the four interactions with the first two pulses, being part of the 'true' nonlinear response of the sample.

We agree with the referee, by no means we intend to discuss incoherent mixing as a main reason to introduce the two-particle description of non-linear response. Notice also that in our terminology, in the same line of refs 29 and 34, "two-particle" refers to "two-chromophores" (not two-excitons) and therefore a two-particle description is the natural consequence of the presence of any kind of inter-chromophore interaction. We changed the second part of the abstract to clarify that the two representations are not motivated by the presence of incoherent mixing:

"In this work, we elaborate on the relation between the non-linear response and the incoherent mixing contribution by analyzing the action signal in terms of one- and two-particle observables."

Regarding the non-generality of the identification of 'non-linear response vs incoherent mixing' with 'multi-particle vs single-particle picture', the referee is right. We have modified the discussion by explicitly commenting on the specific conditions in which the one-particle representation relates to incoherent mixing. At page 17:

"Beyond the weak-coupling regime, energy splitting and dipole redistribution related to excitonic delocalization on the two chromophores must be considered. In this case, Feynman diagrams contributing to cross peak positions (Fig. 3b,d-f) no longer represent the product of one-particle signals. As a result, they do not generate incoherent mixing but rather become expression of the non-linear response of the molecular dimer.[26-28] Therefore, while incoherent mixing of one-particle signals can be always recast as the net contribution of fourth-order pathways in the two-particle picture, the factorization of non-linear pathways into the product of one-particle signals does not hold in general."

Moreover, the argument developed in this paper can be generalized to incoherent mixing involving linear signals of units possibly made of more than one chromophore, breaking again the identification between incoherent mixing and single-particle picture. We only mention such a generalization at an intuitive level to avoid possible confusion with the simpler setting we adopt in the body of the argument. At page 18:

"In light of these considerations, the analysis can be generalized to supramolecular complexes, e.g., LH2 complex, composed of weakly coupled domains, e.g., B800 and B850 rings, interacting only during the detection-time. In this case, both the non-linear response of each domain and the incoherent mixing between different domains can contribute to the signal, eventually overlapping in the spectrum."

2) Drawing the line based on 'system of interest' (p. 18) seems somewhat arbitrary.
How does one draw a line between systems that can still 'mix' within their 'true' nonlinear response, and those who mix 'incoherently'? This should be based at least on some separation of timescales, compared to the time it takes to produce the incoherent signal. However, even this seems difficult – would then what is termed 'incoherent mixing' in fluorescence-detected 2DES be 'true nonlinear response' under photocurrent detection?

We agree with the referee that separation of timescales is a key to define incoherent mixing signal. In our setting, one can refer to incoherent mixing only when the mixing dynamical process, like EEA, is slow enough to not affecting the dynamics during the pulse train. When EEA affects the dynamics probed by the pulse sequence, like in the case of a fast EEA in a six-wave mixing experiment, it is definitely a dynamical process contributing the non-linear response and it must be included as such. Because the timescale separation is between the excitation sequence and the mixing dynamics, the nature of the incoherent signal does not

change the definition of incoherent mixing. But it surely play a role in the final intensity of these contributions, in analogy with the time-gating of the signal mentioned by the referee in a following point. To clarify the importance of timescale separation we add, at page 17:

"This leads to the central issue of how to identify cross peaks representing incoherent mixing. We remark the two conditions necessary to derive the one-particle representation of the signal (Eq. 9), namely weak coupling between chromophores and the timescale separation between the dynamics during the delay-times and the slower mixing process during the detection-time. The latter condition points out that time-gating strategies[26,28,30] can be used to reduce the contribution of incoherent mixing to the spectrum. Indeed, as reported in the Supporting Information, the time-gated signal shows how the term related to incoherent mixing grows as the integration window increases."

3) The terminology of 'incoherent mixing' is deceptive.

I realize that the authors did not invent this term, but their manuscript presents an opportunity to clarify it. In its original use, the term was probably supposed to contrast with the 'coherent' nonlinear response. However, as the authors point out, this is to a large degree just a matter of representation. Incoherent mixing would, by definition, imply mixing without preserving the phase. However, the mixed populations do preserve the combined phase, that is why they are extracted together with the nonlinear response signal.

We agree with the referee and integrate explicitly this point. At page 16:

"In this respect, the term ``incoherent mixing" may be deceptive. Indeed, the mixing signal inherits and preserves the phase combination of the fourth-order interaction sequence, and for this reason, it is extracted together with the non-linear response."

4) Possible better distinction between mixing and nonlinear response?

The authors provide ideas how to distinguish the 'incoherent mixing' and 'nonlinear response'. In light of points 1) and 2) and what authors write on page 18 about the choice of representation, would it be possible to establish a general, system-independent definition of 'nonlinear response' and 'signal mixing'. Would, as a suggestion, simply defining 'whatever happens between the pulses' as 'nonlinear response', and 'whatever happens during signal emission' as 'signal mixing'?

We believe we cannot establish a general, system-independent definition of 'nonlinear response' and 'signal mixing'. Instead, we have defined the general conditions (actually pertaining the system) under which some components of the non-linear response assume the character of incoherent mixing (see point 2 above). The suggestion of the referee is certainly valid in the sense that if the mixing dynamics does not happen, no incoherent mixing will appear. However, there are slow dynamics during the detection time which certainly influences the resulting spectra but cannot be tagged as "incoherent mixing". An example is when the weak

coupling condition between the excitations breaks down, like in the biexciton state in nanocrystals. In that case, the relatively slow recombination of the biexciton via Auger relaxation does influence the spectrum but cannot be considered as incoherent mixing. Indeed the resulting spectral features does not resemble the product of single-exciton transitions.

5) The 'incoherent mixing' can be distinguished and probed by gating the signal.
Several works, including that of the authors (references 25, 35 of the manuscript) have studied dependence of the mixing (typically in the form of spectral cross peaks) on the time of the signal emission. Indeed, if one would gate the signal immediately after the interaction with the last pulse, there should be no time for nonlinear population mixing, and the signal should consist of the nonlinear response only. (This motivates the proposed definition in point 5). The authors could comment on the possibility of signal gating in manipulating the incoherent mixing part of the signal.
We agree with the reviewer about the possibility of exploiting time-gating to reduce the effect of incoherent-mixing. The reference to time-gating has been included in the main text (see answer to point 2).

Additional Questions:
Urgency: Top 10%

Significance: Top 10%

Novelty: High

Scholarly Presentation: Top 10%

Is the paper likely to interest a substantial number of physical chemists, not just specialists working in the authors' area of research?: Yes

Reviewer: 2

Recommendation: This paper is publishable subject to minor revisions noted. Further review is not needed.

Comments:

The manuscript deals with interpretational issues of the action detected 2D electronic spectroscopy. The action-detected coherent spectroscopy combines the advantages of fully coherent methods with the possibly higher detection sensitivity of the action detection schemes such as fluorescence detection. Fluorescence-detected 2D electronic spectroscopy ideally reports on the same processes and electronic structure relations as the coherently detected version of the method, however, it has been shown that exciton-exciton annihilation deletes certain signal components. Most notably, there are cross-peaks at waiting time T=0 in the fluorescence-detected spectra of a weakly coupled dimer, while no cross-peaks appear in the coherently detected spectra.

The cross-peaks in the action detected 2D spectra caused confusion in some early publications and there were various claims about them reporting interactions between molecules in the same way as the coherently detected methods. One reason for this confusion was apparent alternative explanations of the origin of the signals occurring in the method. The present paper attempts to reconcile the alternatives and show that in the weak coupling limit, they provide equivalent descriptions.

I have to admit that I come completely from the non-linear response side of the story. Non-linear response theory explains all the signals in both detection versions of the spectroscopy and does that both in the weak and strong interaction limits. This is the reason why I always hesitated to spend extensive time learning the background of the incoherent mixing description. The value of the present paper for me is in bringing closer to attention some of the advantages of the alternative. The authors make a good job of comparing the two descriptions, pointing out their similarities and differences. It is important that they link the two descriptions with the one-particle and two-particle descriptions. From this linking it is immediately clear that there might be practical advantages in treating weakly coupled systems with the incoherent mixing picture.

We thank the reviewer for pointing out so clearly why a rather abstract point like formulating the response in one or two-particle representations is also practically relevant. On a side note, we completely agree with the reviewer that the non-linear response theory is the most general description, and we explicitly include this consideration in the revised manuscript (see answer to point 1 of referee 1)

I have no doubt that this paper is a valuable addition to the literature, clarifying an important interpretational issue and also pointing towards its practical combination with non-linear response theory. I think the paper can be published with the minor adjustments that I suggest below.

I have two suggestions:

1) The paper is overall well-written and structured. At a few points, the wording isn't very clear and I suggest that the authors check their English again to make the text more clear.

Some sentences have been rephrased for clarity.

2) I suggest that the authors use their insight into both alternative explanations of the signals to clarify a seemingly unimportant etymological issue with the term incoherent mixing. I explain briefly, why I think this is important: The initial confusion about the nature of the cross-peaks might have been related to the fact that the word "mixing" has at least two meanings within the spectroscopy of excitons. Cross-peaks in the coherent 2D spectra report on "excitonic-mixing", i.e. the fact that the observed states are superpositions of local states. This is often interpreted as the presence of coherence and hence it is the "coherent mixing". Clearly the term "incoherent mixing" suggests very much some different type of mixing of exciton states, and I believe that in some early publications (or attempted publications) this idea was used. Nevertheless, there is a different meaning of "mixing" also at play, and this is the mixing of signals, such as in "four-wave mixing". The mixing of signals has nothing to do with the properties of the studied molecular system. Now, which of the two meanings, if any, is closer to the nature of the incoherent mixing discussed in this paper? Clearly, incoherent mixing is not a mixing of excitonic states as it stems from the processes occurring during the detection time. It would be very useful to the reader (and this referee) if the authors could make a comment in the paper on the nature of incoherent mixing along these lines.

In the revised discussion, we elaborate more explicitly on a few points related to the nature of incoherent mixing signal: first, we made clearer that incoherent mixing cannot occur beyond the weak coupling limit, thus excluding its relation with any form of excitonic mixing (see also answer to points 1 and 2 of referee 1). The character of the incoherent mixing phenomenon is indeed a mixing of signals, as it is generated by a net contribution of non-linear pathways factorizable into lower order one-particle signals. We remark this definition at page 16-17:

"Beyond the weak-coupling regime, energy splitting and dipole redistribution related to excitonic delocalization on the two chromophores must be considered. In this case, Feynman diagrams contributing to cross peak positions (Fig. 3b,d-f) no longer represent the product of one-particle signals. As a result, they do not generate incoherent mixing but rather become expression of the non-linear response of the molecular dimer.[26-28]"

From the etymological point of view, we tend to associate the "incoherent" character of the incoherent mixing signal to its lack of rephasing behaviour, as specify at page 17 of the revised manuscript:

"Recognizing the presence of incoherent mixing is especially important because the associated spectral features may hide relevant spectral dynamics. Since in the weak coupling regime, the environments of the two chromophores can be considered as independent, cross-population pathways do not have rephasing capability.[41] As a result, incoherent mixing contributions are not diagonally elongated and their lineshape is not expected to undergo significant changes along the waiting-time T_2.[31]"

However, we notice that in this context also point 3 of Reviewer 1 is certainly meaningful.

Additional Questions:

Urgency: High

Significance: Top 10%

Novelty: High

Scholarly Presentation: High

Is the paper likely to interest a substantial number of physical chemists, not just specialists working in the authors' area of research?: Yes
